# A ubiquitous spectrolaminar motif of local field potential power across the primate cortex

**DOI:** 10.1038/s41593-023-01554-7

**Published:** 2024-01-18

**Authors:** Diego Mendoza-Halliday, Alex James Major, Noah Lee, Maxwell J. Lichtenfeld, Brock Carlson, Blake Mitchell, Patrick D. Meng, Yihan (Sophy) Xiong, Jacob A. Westerberg, Xiaoxuan Jia, Kevin D. Johnston, Janahan Selvanayagam, Stefan Everling, Alexander Maier, Robert Desimone, Earl K. Miller, André M. Bastos

**Affiliations:** 1 McGovern Institute for Brain Research, Massachusetts Institute of Technology, Cambridge, MA USA; 2 The Picower Institute for Learning and Memory, Massachusetts Institute of Technology, Cambridge, MA USA; 3 Department of Psychology, Vanderbilt University, Nashville, TN USA; 4 Vanderbilt Vision Research Center, Vanderbilt University, Nashville, TN USA; 5 Vanderbilt Brain Institute, Vanderbilt University, Nashville, TN USA; 6 Department of Vision and Cognition, Netherlands Institute for Neuroscience, Royal Netherlands Academy of Arts and Sciences, Amsterdam, The Netherlands; 7 School of Life Sciences, Tsinghua University, Beijing, China; 8 IDG/McGovern Institute for Brain Research, Tsinghua University, Beijing, China; 9 Graduate Program in Neuroscience, Western University, London, ON Canada; 10 Centre for Functional and Metabolic Mapping, Robarts Research Institute, University of Western Ontario, London, ON Canada; 11 Department of Physiology and Pharmacology, University of Western Ontario, London, ON Canada

**Keywords:** Neural circuits, Sensory processing, Electrophysiology

## Abstract

The mammalian cerebral cortex is anatomically organized into a six-layer motif. It is currently unknown whether a corresponding laminar motif of neuronal activity patterns exists across the cortex. Here we report such a motif in the power of local field potentials (LFPs). Using laminar probes, we recorded LFPs from 14 cortical areas across the cortical hierarchy in five macaque monkeys. The laminar locations of recordings were histologically identified by electrolytic lesions. Across all areas, we found a ubiquitous spectrolaminar pattern characterized by an increasing deep-to-superficial layer gradient of high-frequency power peaking in layers 2/3 and an increasing superficial-to-deep gradient of alpha-beta power peaking in layers 5/6. Laminar recordings from additional species showed that the spectrolaminar pattern is highly preserved among primates—macaque, marmoset and human—but more dissimilar in mouse. Our results suggest the existence of a canonical layer-based and frequency-based mechanism for cortical computation.

## Abstract (web summary)

This study reports a motif of local field potentials that maps onto the anatomical layers of the cortex, is preserved across macaque cortical areas and across primates and may represent a ubiquitous layer-based and frequency-based cortical mechanism.

## Main

One of the most prominent structures of the mammalian brain is the cerebral cortex, which is thought to underlie complex cognitive functions. Despite the vast diversity of functions carried out by different areas of the cortex, almost all areas share a ubiquitous anatomical motif composed of six layers, with relatively minor variations^1^. This observation has led to the hypothesis that all cortical areas are composed of a common canonical microcircuit that is the fundamental unit for computation^2–4^; by understanding the principles of the canonical microcircuit, one should be able to explain how all areas of cortex accomplish their functions with variations of the ubiquitous laminar motif. This hypothesis has inspired many theoretical proposals of cortical function^5–7^.

It is reasonable to hypothesize that the anatomical differences between cortical layers in cell size, composition and projections give rise to distinct laminar activity patterns. Because the overall laminar anatomical motif^8,9^ is relatively preserved across cortical areas and across individual subjects, the corresponding laminar activity patterns should also be preserved across cortical areas and subjects. Moreover, in all areas and subjects, the activity patterns should consistently map onto the same anatomical landmarks of the laminar architecture.

Numerous studies have observed laminar activity patterns^10–18^. However, these patterns have been observed in a given cortical area and in the context of a given function, not as a common phenomenon across cortex. It has been proposed that there is a canonical laminar activation pattern that reflects the ubiquitous anatomical laminar motif of the cortex: an initial excitation in layer 4, followed by subsequent excitation in layers 2/3 and then layers 5/6 (refs. ^4,19^). Using current source density (CSD) analysis of local field potentials (LFPs)^20^, this activation pattern has been observed in visual cortex and is currently the established method for estimating the relative location of cortical layers in electrophysiological recordings^21,22^. However, the generality of this circuitry has been questioned by the observation that deep layers can be activated independently of superficial layers^23^. Furthermore, the CSD pattern is driven by sensory input and, thus, may be less common in non-sensory areas.

It has also been proposed that cortex generates a canonical laminar activity pattern composed of gamma rhythms (50–150 Hz) in superficial layers and alpha-beta rhythms (10–30 Hz) in deep layers^5,10,12,15,18,24–29^. However, other reports have stressed distinct laminar activity patterns in the inferotemporal (IT) cortex^11^ and the supplementary eye field (SEF)^28^ compared to early visual cortex in macaque cortex. If such a canonical pattern of superficial-layer gamma and deep-layer alpha-beta exists, it could provide a scaffold for these rhythms to functionally segregate feedforward and feedback inter-areal communication, respectively^18,30–33^.

**Fig. 1:**
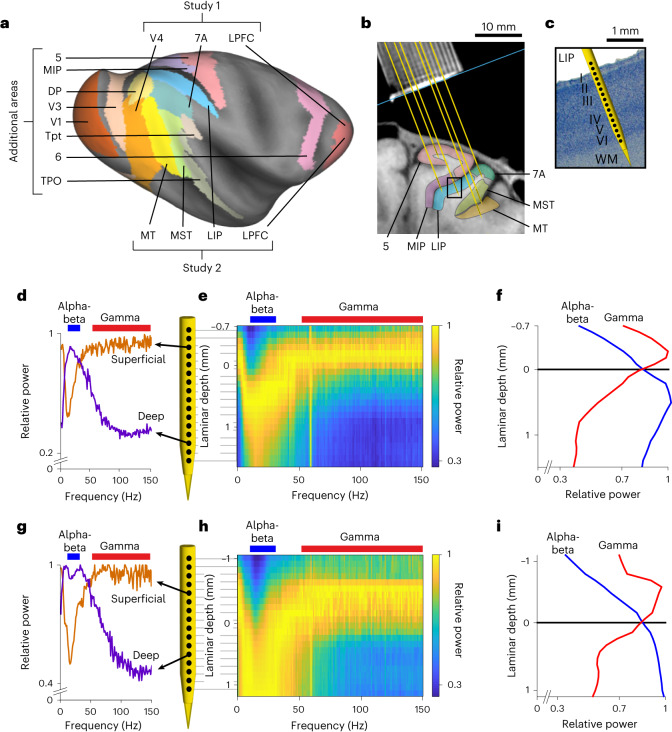
Laminar recording methods and laminar differences in LFP oscillatory power. **a**, Inflated cortical surface of the macaque brain showing cortical areas recorded depicted using Caret software^60^ on the F99 template brain and using Lewis and van Essen^61^ area parcellation scheme. **b**, Structural MRI nearly-coronal section of one monkey from study 2 showing recording chamber grid (top) and location of areas MT, MST, 7A, 5, MIP and LIP on the right hemisphere. Yellow lines show the locations of example probes in all areas. **c**, Nissl section from the same monkey corresponding to a ×10 magnification of the black rectangular region in **b** with an example probe diagram showing the locations of recording channels (black dots) with respect to the cortical layers in area LIP. WM, white matter. **d**,**g**, Relative power as a function of frequency in a superficial-layer channel and a deep-layer channel from two example probes in areas LIP (**d**) and MT (**g**). **e**,**h**, Relative power maps for the two example probes. **f**,**i**, Relative power averaged in the alpha-beta (blue) and gamma (red) frequency bands as a function of laminar depth for the two example probes. Laminar depths are measured with respect to the alpha-beta/gamma crossover.

Whether the cortex contains a canonical laminar oscillatory activity pattern, and whether this pattern is preserved across all of cortex, remains unknown. To investigate this, we recorded LFP signals across all cortical layers using multicontact laminar probes. We combined data collected in multiple laboratories from five macaque monkeys and 14 cortical areas spanning a variety of hierarchical processing stages and functions (Fig. 1a): V1 (primary visual cortex), V3, V4, middle temporal (MT) (early visual areas), medial superior temporal (MST) (a visual association and multimodal area), medial intraparietal (MIP) (a visual/somatosensory/motor area), area 5 (somatosensory cortex), area 6 (premotor cortex), dorsal prelunate (DP), Tpt (temporo-parietal-auditory cortex), TPO (temporo-parieto-occipital junction; a polysensory area), 7A, lateral intraparietal (LIP) (higher-order parietal association areas) and lateral prefrontal cortex (LPFC) (a higher-order executive area). Across all areas, we observed a common laminar pattern, which we termed the spectrolaminar motif: LFP power in the gamma frequency band (50–150 Hz) was strongest in superficial layers, and the alpha-beta band (10–30 Hz) was strongest in deep layers.

To test whether the spectrolaminar motif consistently aligns with specific anatomical layers in macaques, we performed electrolytic lesions. Histological analyses revealed that key landmarks of the spectrolaminar motif consistently mapped onto the same anatomical layers: peak gamma power was located in layers 2/3; peak alpha-beta power was located in layers 5/6; and the crossover between relative gamma and alpha-beta power corresponded to layer 4.

Finally, we tested whether the spectrolaminar motif generalizes across other species by analyzing laminar recordings from marmoset, human and mouse cortex. The spectrolaminar pattern was highly similar among the macaque, marmoset and human but was qualitatively and quantitatively more dissimilar between these primates and mice.

## Results

The aim of this study was to investigate whether oscillatory neuronal activity represented in the LFPs differs between cortical layers and, if so, whether the laminar activity pattern is preserved across cortical areas and species. To answer this, we first analyzed LFPs from intracortical electrophysiological recordings performed in multiple cortical areas of rhesus macaque monkeys using multicontact laminar probes (16, 24 or 32 contacts). We first combined data from six cortical areas collected in two independent studies performed in different laboratories. Additional data were also collected from eight other areas. The 14 areas in the combined dataset varied broadly in their anatomical and hierarchical position, ranging from V1 to LPFC (Fig. 1a). The probes were positioned with guidance from structural magnetic resonance imaging (MRI) so that the contacts (that is, recording channels) traversed all cortical layers as perpendicularly to the cortical sheet as was possible given the orientation of each cortical area with respect to the recording chambers (Fig. 1b,c). The relative position of each probe’s channels with respect to cortex was confirmed by assessing the presence of multi-unit activity. The two studies used different behavioral tasks (Methods). In both tasks, trials began with a period of gaze fixation followed by a visual stimulation (presentation of a static picture in study 1 and a moving random dot surface in study 2). Our analyses were applied to signals collected in the fixation and sensory stimulation periods.

### Spectrolaminar motif of LFP power in the macaque cortex

To compare the oscillatory activity of the LFP signals between cortical layers recorded by each probe, we obtained, for each channel, the mean LFP power spectrum across trials during the fixation and sensory stimulation periods of the task; at each frequency, we then divided the power of each channel by that of the channel with the highest power. The resulting relative power spectrum of individual channels revealed a common pattern across probe recordings: LFP power in the gamma frequency band (50–150 Hz) was higher in superficial channels, whereas power in the alpha-beta frequency band (10–30 Hz) was higher in deep channels (Fig. 1d,g). This observation suggested the possibility that the relative power varies smoothly across cortical layers and frequencies. To examine this, we stacked the relative power spectra of all channels in each probe to create a two-dimensional frequency-by-depth matrix of relative power values with a size of 150 1-Hz bins by 32 channels, referred to as the relative power map (Fig. 1e,h and Methods).

**Fig. 2:**
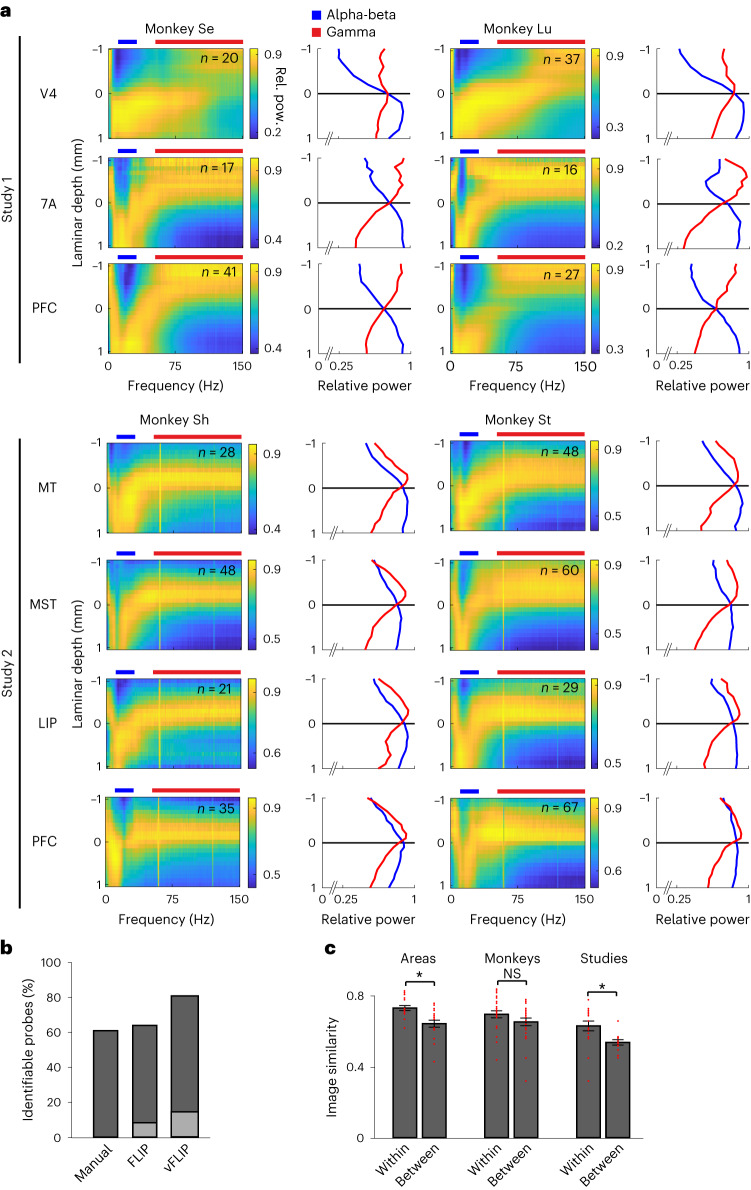
The spectrolaminar pattern is ubiquitous across areas, monkeys and studies. **a**, For each cortical area in each monkey and each study, across-probes mean relative power map (left) and mean relative power in the alpha-beta (blue) and gamma (red) bands as a function of laminar depth with respect to the alpha-beta/gamma crossover channel (right). **b**, The percentage of probes with an identifiable alpha-beta/gamma crossover using different identification methods: manual, FLIP and vFLIP. Light gray bars, percentage of identifiable probes after shuffling channel positions. **c**, Mean IS across all comparisons within area (*n* = 14), between areas (*n* = 18), within monkey (*n* = 32), between monkeys (*n* = 25), within study (*n* = 18) and between studies (*n* = 12). Data points for all comparisons are shown. Error bars, mean ± s.e.m. across all (independent) comparisons. Two-tailed unpaired *t*-tests were used to compare within versus between areas (*P* = 0.0029), within versus between monkeys (*P* = 0.15) and within versus between studies (*P* = 0.019). *, significant difference; NS, not significant.

The relative power maps confirmed a smoothly varying transition of power across channel depths and frequencies, forming a characteristic spectrolaminar motif resembling a radical sign: the peak relative power (yellow tones in Fig. 1e,h) shifted from superficial channels at delta-theta frequencies (1–8 Hz) to deep channels at alpha-beta frequencies and back to superficial channels at gamma frequencies. To better examine how power at different frequency bands varies across layers, we averaged the relative power across the delta-theta, alpha-beta and gamma bands as a function of depth. We found an increasing deep-to-superficial gamma power gradient peaking in superficial channels and an increasing superficial-to-deep alpha-beta power gradient peaking in deep channels (Fig. 1f,i). We tested whether these opposing LFP power gradients were present in all areas. Combining all monkeys and areas from study 1 and study 2, our dataset consisted of 810 probe recordings. Opposing gradients of alpha-beta and gamma relative power were identifiable in 61% of the probes using a manual method, in 64% using frequency-based layer identification procedure (FLIP)—a fully automated algorithm that we developed—and in 81% using vFLIP, a frequency-variable version of FLIP (Figs. 2b and 5, Extended Data Fig. 8b, Methods and the ‘FLIP’ subsection in ‘Results’). Notably, the percentage of identifiable probes was low if channel positions were shuffled within-probe to destroy laminar information (Fig. 2b).

**Extended Data Fig. 1:**
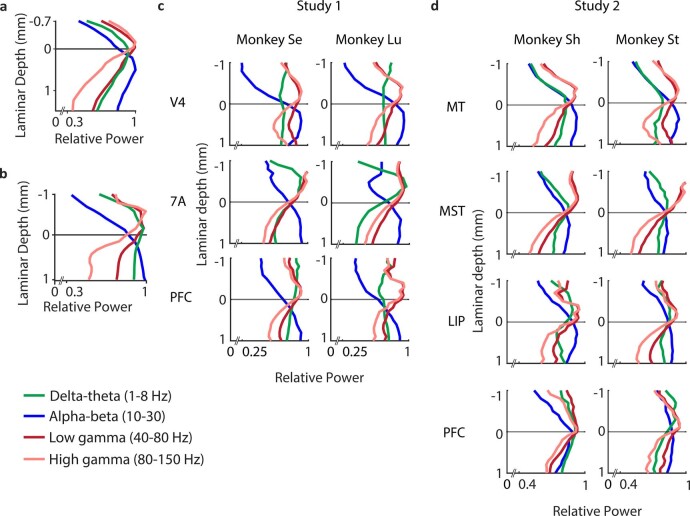
Laminar relative power across different frequency bands. Relative power in the delta-theta (green line), alpha-beta (blue), low gamma (dark red), and high gamma (pink) frequency bands as a function of laminar depth for two example probes (**a**, **b**), and averaged across probes from each area and monkey in Study 1 (**c**) and Study 2 (**d**).

To examine how consistent the spectrolaminar motif was across individual probes in each area, we aligned the relative power maps of all individual probes by the alpha-beta/gamma crossover channel (the channel at which the relative power of alpha-beta and gamma are equal), and we then averaged the relative power maps across probes for each cortical area in each monkey and each study (Fig. 2a). All mean relative power maps showed the presence of a spectrolaminar motif similar to the individual examples, characterized by higher gamma power (and, to a lesser extent, delta-theta power; Extended Data Fig. 1) in superficial channels than deep ones and higher alpha-beta power in deep channels than superficial ones. An increasing deep-to-superficial power gradient was present for the lower gamma frequencies (40–80 Hz) to a similar extent as for the higher gamma range (above 80 Hz), indicating that this gradient is not due to contamination of the LFP by spiking activity (Extended Data Fig. 1). That the spectrolaminar motif is clearly visible in the mean relative power maps, and that these maps are similar among areas, monkeys and studies, strongly suggest that the motif is a ubiquitous property across cortex.

### Spectrolaminar motif across areas, monkeys and studies

To quantify the degree of similarity between the relative power maps of probes recorded within or between cortical areas, monkeys and studies, we expressed each relative power map as a bi-dimensional frequency-by-depth image and applied image similarity (IS) analysis—an image-computable metric that quantifies the similarity between two images from 0 (most dissimilar) to 1 (identical)^34^. We grouped probes by cortical area, and, for each pair of areas within and between monkeys and studies, we obtained the IS value comparing the across-probes mean relative power maps within or between groups using a randomized probe subgrouping procedure (Methods).

**Extended Data Fig. 2:**
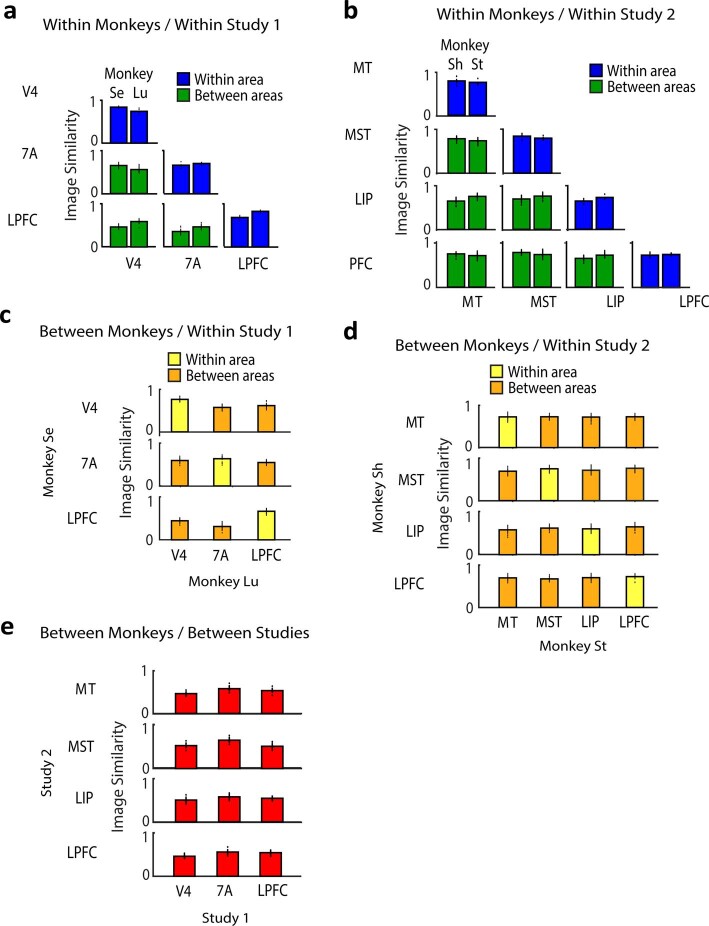
Image similarity values between relative power maps within and between cortical areas, monkeys, and studies. (**a**, **b**) Mean image similarity of relative power maps across probe recordings within (blue) and between (green) areas within each monkey within Study 1 (a) and Study 2 (b). (**c**, **d**) Mean image similarity within (yellow) and between (orange) areas between monkeys within Study 1 (c) and Study 2 (d). (**e**) Mean image similarity between areas, between monkeys and between studies. In all panels, the height of each bar corresponds to the mean image similarity across all randomized probe splits (shown as individual dots; see Methods). In all panels, n = 5 probe splits for within-area comparisons, and n = 20 probe splits for between-areas comparisons.

Although all cortical areas share a common anatomical laminar motif, it is well known that this motif shows variations between areas. Therefore, we considered the possibility that the spectrolaminar motif also varies across areas. To assess this, we compared the IS values of relative power maps recorded within and between different areas within monkeys. We found that mean IS across all within-area comparisons was significantly higher than mean IS in between-area comparisons (Fig. 2c and Extended Data Fig. 2). This suggests that, despite the similarity in the spectrolaminar pattern between cortical areas, each area differs from others to a small degree. Next, we compared the similarity of relative power maps recorded from the same monkey versus from different monkeys and found no significant difference (Fig. 2c and Extended Data Fig. 2). This suggests that the spectrolaminar motif is consistent across individual monkeys and that inter-individual differences are minor. Finally, we found that the similarity of relative power maps recorded within each study was significantly higher than between studies (Fig. 2c and Extended Data Fig. 2). Thus, despite the generalization of the spectrolaminar patterns across studies, the patterns are most similar when recorded by the same study.

**Fig. 3:**
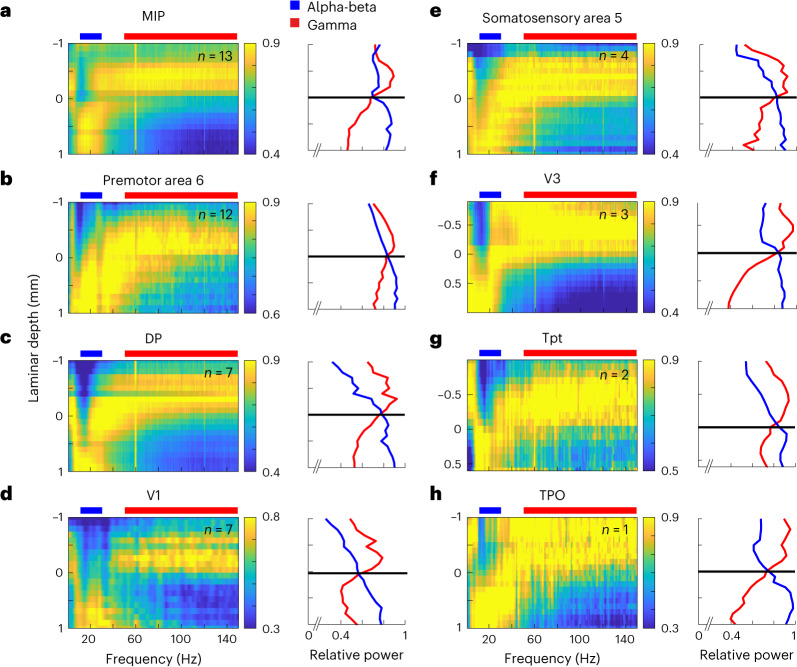
Spectrolaminar pattern in eight additional areas. **a**–**h**, For each cortical area, across-probes mean relative power map (left) and mean relative power in the alpha-beta (blue) and gamma (red) bands as a function of laminar depth with respect to the alpha-beta/gamma crossover channel (right). Number of probes averaged is indicated on the left subplot.

To further confirm the ubiquity of the spectrolaminar motif beyond the six areas recorded in study 1 and study 2, we performed recordings in eight additional cortical areas that varied in their degree of lamination (from highly laminated to dysgranular—that is, lacking layer 4) and cortical system (motor, somatosensory and auditory), including V1, V3, DP, somatosensory area 5, premotor area 6 (PMd; a dysgranular area), auditory area Tpt, polysensory area TPO and polysensory/somatomotor MIP. The spectrolaminar motif was present in each of these areas (Fig. 3).

### Histological mapping of the spectrolaminar motif

Having established that the spectrolaminar motif was present in at least 14 cortical areas (Figs. 1–3), we hypothesized that the pattern is anchored to specific anatomical layers and that this correspondence is consistent across cortical areas. Alternatively, it could be that the spectrolaminar motif is present in each area, but, given the laminar variation between areas, it does not consistently correspond to specific layers. To test this, we parametrized the spectrolaminar motif using three electrophysiological markers: the two probe channels having the highest power in the gamma and in the alpha-beta frequency ranges and the channel at which the relative power of gamma and alpha-beta was equal (that is, the crossover).

**Fig. 4:**
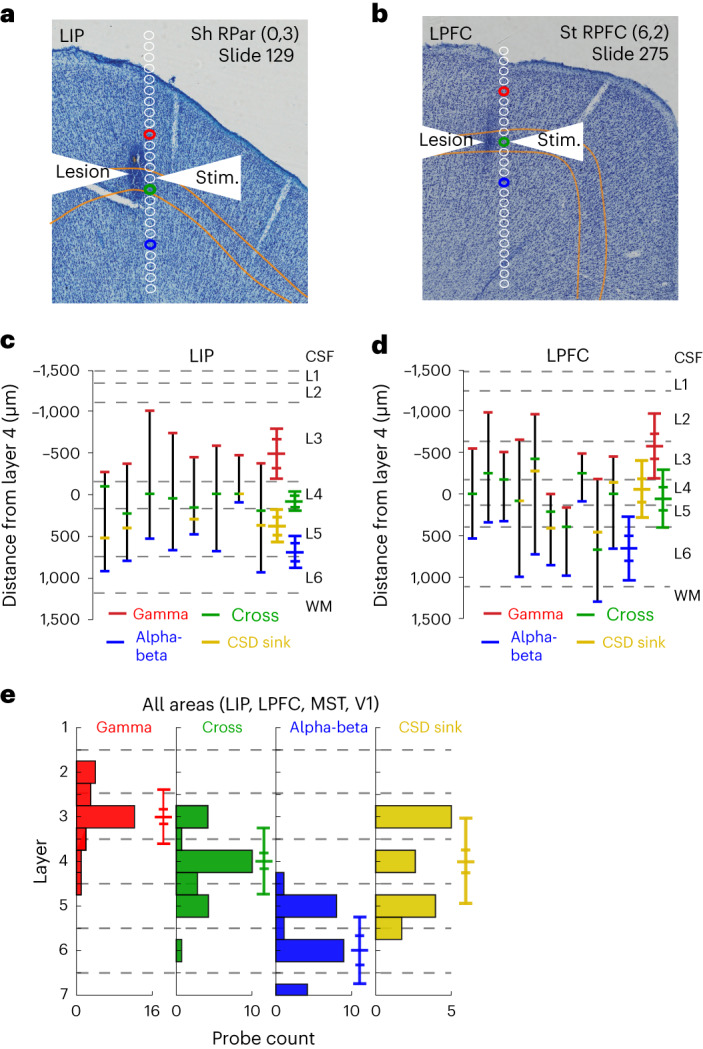
Histological mapping of spectrolaminar motif of relative LFP power with respect to anatomical layers. **a**, Example histological Nissl-stained section in area LIP in monkey Sh showing a clear electrolytic lesion (dark spot; see white arrows). Reconstructed probe channels are shown in white. The laminar position of layer 4 is outlined in yellow. The red, green and blue dots correspond to the channel with highest gamma power, the alpha-beta/gamma crossover and the channel with highest alpha-beta power on the probe. **b**, Same as **a** but for area LPFC in monkey St. **c**, For each independent probe in area LIP (*n* = 8), we performed probe reconstructions shown in **a** and **b** and then measured the distance from each physiological landmark (gamma peak power in red, alpha-beta peak power in blue, crossover in green and CSD sink in yellow) to the center of layer 4 in micrometers. Each black line is an independent probe. The mean ± s.e.m. and s.d. are indicated with horizontal colored lines. Gray dashed lines indicate the mean laminar boundaries for that area. **d**, Same as **c** but for area LPFC (*n* = 10). **e**, Histograms of the layers where the four physiological measures were found across all available data (LIP, LPFC, MST and V1, *n* = 23 probes for Gamma/Alpha-beta/Cross, and *n* = 14 probes for CSD sink). Median ± 95% CI and s.d. are indicated with horizontal colored lines. CSF, cerebrospinal fluid; L, layer; RPar, right parietal cortex tissue block; RPFC, right lateral prefrontal cortex tissue block; WM, white matter.

**Extended Data Fig. 3:**
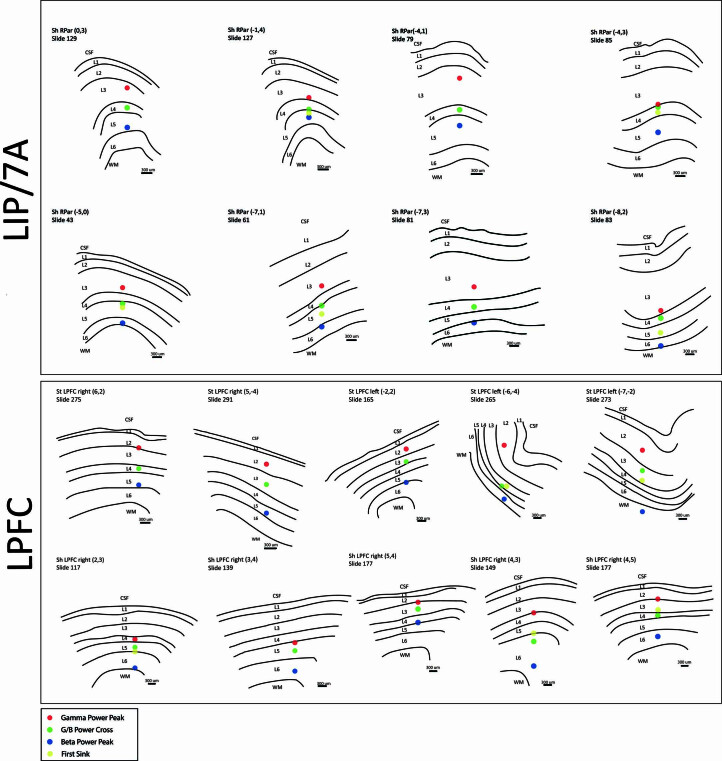
Individual anatomical probe reconstructions for parietal (LIP/7 A) and lateral prefrontal cortex. *Upper panel*, traces of individual brain slices are shown for areas LIP/7 A with anatomically-defined layers labelled from cerebrospinal fluid (CSF), layer 1–6 (L1–L6), and white matter (WM). Each example includes monkey name, brain region, probe grid location, and histological slice number. Red, green, blue, and yellow dots correspond to gamma peak, relative power cross-over, alpha-beta peak, and CSD early sink respectively. *Lower panel*, traces of individual brain slices in LPFC.

**Extended Data Fig. 4:**
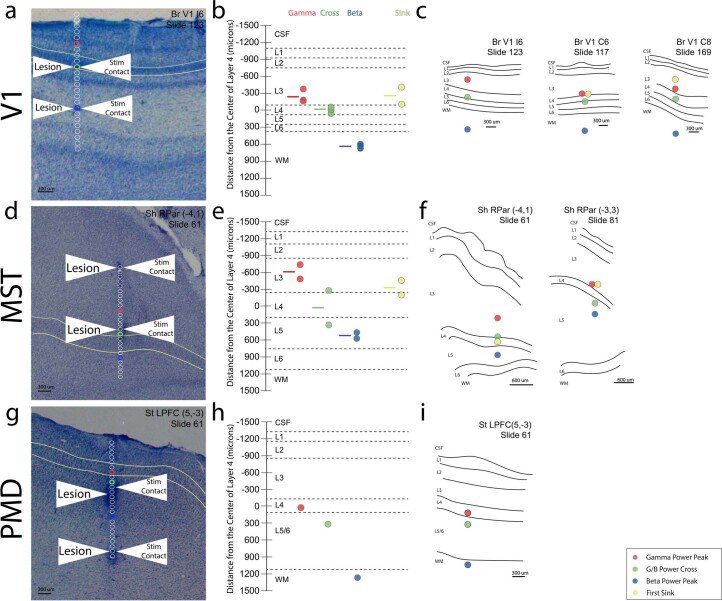
Histological results and examples from V1, MST, and PMD. (**a**) Example histological slice from V1. Identification of electrolytic lesions allows reconstruction of all channels of the laminar probe, shown as open circles. Red circle represents probe contact with gamma power peak, green circle represents cross-over, and blue circle represents alpha-beta peak. Green curves lines demarcate layer 4. (**b**) Population results from V1 histological reconstruction (n = 5). Gamma peak (red), cross-over (green), alpha-beta peak (blue), and CSD early sink (yellow) are shown as distances from center of anatomically-determined layer 4. Negative values indicate more superficial locations, towards layer 1 and CSF (cerebrospinal fluid). (**c**) Individual anatomical probe reconstructions for primary visual cortex. (**d**–**f**) Example histological slices and reconstructed electrophysiological results from middle superior temporal area (MST). (**g**–**i**) Example histological slice and reconstructed electrophysiological results from dorsal premotor cortex (PMD). Scale bars are indicated in each subplot with black horizontal lines.

For a subset of areas (LIP, LPFC, MST, V1 and PMd), we performed additional electrophysiological recordings during which we created electrolytic markers to precisely reconstruct the probe’s location in histological sections (*n* = 8 probe locations in LIP, *n* = 10 in LPFC, *n* = 2 in MST, *n* = 3 in V1 and *n* = 1 in PMd; Methods). Subsequently, we performed histological analysis of the brain tissue (Methods). Example Nissl stains from two recording sessions in areas LIP and LPFC containing electrolytic markers are shown in Fig. 4a,b. The electrolytic marker can be identified in the Nissl section as a circular spot darker than the surrounding tissue. The locations of all channels in the probe were then reconstructed relative to the electrolytic marker by accounting for the known inter-channel spacing and tissue shrinkage due to histological processing (Methods). The colored dots correspond to the channel with highest gamma power (in red) and alpha-beta power (in blue) and the crossover channel (in green). In both examples, the channel with highest gamma power was in layer 3; the channel with highest alpha-beta power was in layer 5; and the crossover was in layer 4 or the layer 4-to-5 boundary. Extended Data Fig. 3 shows each individual probe reconstruction with physiological and laminar landmarks in LIP and LPFC. Probe reconstructions with histology for areas with fewer data points (V1, MST and PMd) are shown in Extended Data Fig. 4. The locations of gamma in superficial layers and alpha-beta in deep layers were largely consistent for these areas, but V1 alpha-beta was an exception. V1 had the highest alpha-beta power in white matter channels, likely due to volume conduction of signals from the cortical sheet of area V2 across white matter due to its exceptional proximity.

To quantify these results and test their robustness, we measured the distance of each physiological marker to the center of layer 4 in micrometers. Negative values indicate that the physiological marker was more superficial than layer 4. Positive numbers indicate that the physiological marker was deeper than layer 4. The results are shown in Fig. 4c,d. Each line indicates independent measurements from separate probes. The mean distance of the crossover channel to layer 4 was 58 μm for LIP and 27 μm for LPFC (95% confidence interval (CI) (−19 μm, 135 μm) for LIP and (−183 μm, 237 μm) for LPFC).

These measured distances to the middle of layer 4 could, in principle, correspond to different layers because layer thickness across cortex is variable^35^. Therefore, we reconstructed the physiological markers for each probe and quantified the average layer at which they were observed. If markers were observed at a border between layers, they were counted as a value of 0.5 away from the layer center. For example, a physiological marker at the border between layers 2 and 3 was given a value of 2.5. We performed this analysis after collecting all available data from LIP, LPFC, MST and V1 (Fig. 4e). The median laminar position of the gamma peak was 3 (*n* = 24, 95% CI of the mean (2.63, 3.2)); gamma/beta crossover was 4 (*n* = 24, 95% CI of the mean (3.82, 4.45)); and alpha-beta peak was 6 (*n* = 24, 95% CI of the mean (5.43, 6.05)).

### FLIP

**Fig. 5:**
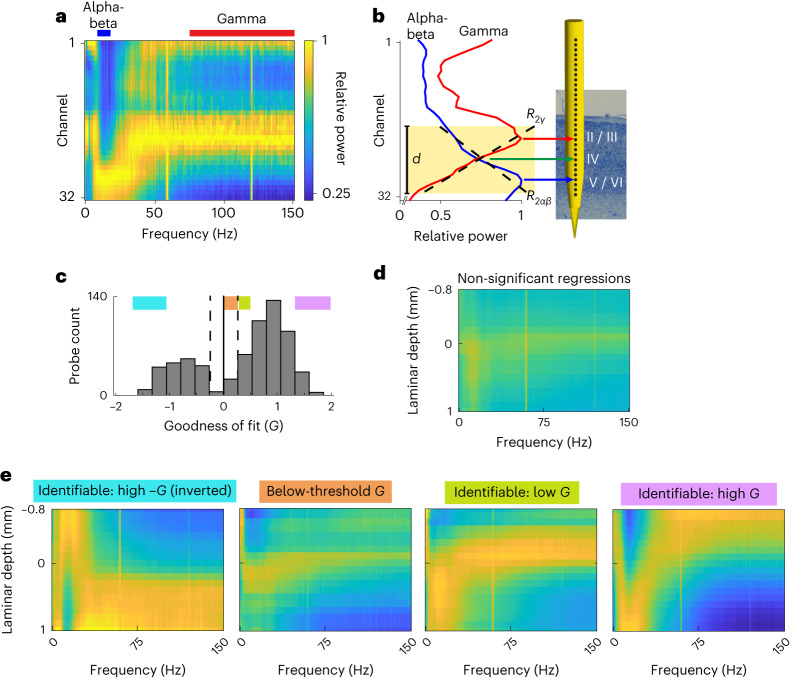
Automatic FLIP. **a**,**b**, FLIP steps for an example probe. First, FLIP automatically computes the relative power map (**a**) and the mean relative power across the alpha-beta (10–19 Hz, blue) and gamma (75–150 Hz, red) optimal bands. Then, it identifies the channel range *d* where the *G* value of the alpha-beta and gamma relative power regressions (dashed black lines) is maximal (**b**). Finally, it identifies the alpha-beta peak (blue arrow), gamma peak (red arrow) and crossover (green arrow) channels as markers for layers 5/6, 2/3 and 4, respectively. **c**, Histogram showing distribution of *G* across all probes. Dashed lines, ±*G* threshold. **d**, Mean relative power map across all probes with non-significant alpha-beta and gamma relative power regressions. **e**, Mean relative power map across probes within each of the four colored ranges of *G* shown in **c**.

Taking advantage of our finding that the spectrolaminar motif accurately maps onto histologically identified cortical layers and is highly preserved across cortical areas, monkeys and studies, we developed a fully automated frequency-based layer identification procedure (FLIP). With no user input, FLIP maps the location of channels in a linear probe with respect to the cortical layers during electrophysiological recordings. It is implemented in MATLAB and is freely accessible (see ‘Code availability’ section), easy to use and fast. The user starts by calculating the LFP power spectrum from the raw electrophysiological data recorded by a laminar probe. FLIP uses as input the LFP power spectrum as a function of frequency for all probe channels individually. Then, it determines the range of consecutive channels *r* that maximizes the goodness of fit (*G*)—a metric that quantifies how well the mean relative power in the alpha-beta (10–19 Hz) and gamma (75–150 Hz) optimal bands across channel depths is fit by linear regressions of opposite slopes with coefficients *R*^2^_*αβ*_ and *R*^2^_*γ*_, respectively (Fig. 5a,b). If both regression coefficients are significant and if *G* exceeds a threshold *G*_*t*_, FLIP considers the probe to have an identifiable spectrolaminar pattern (Methods). If so, FLIP identifies the channels corresponding to the alpha-beta peak, gamma peak and crossover within the range *r* and uses them to map layers 5/6, 2/3 and 4, respectively (Fig. 5b). All other channels are then mapped with reference to those three anatomical landmarks.

Figure 5c shows the distribution of *G* values across probe recordings. As shown in Fig. 5e, *G* accurately estimates the quality of the probes’ spectrolaminar pattern: probes with high *G* show better-resolved patterns than those with low *G*. Probes with at least one non-significant regression coefficient (Fig. 5d) or with below-threshold *G* (Fig. 5c,e) are automatically considered to lack an identifiable spectrolaminar pattern, and their layers cannot be mapped. Furthermore, FLIP identifies an inverted spectrolaminar pattern when the orientation of the cortical sheet is inverted relative to the probe insertion (negative *G*; Fig. 5e), as is the case with MIP and MST (Fig. 1b).

**Extended Data Fig. 5:**
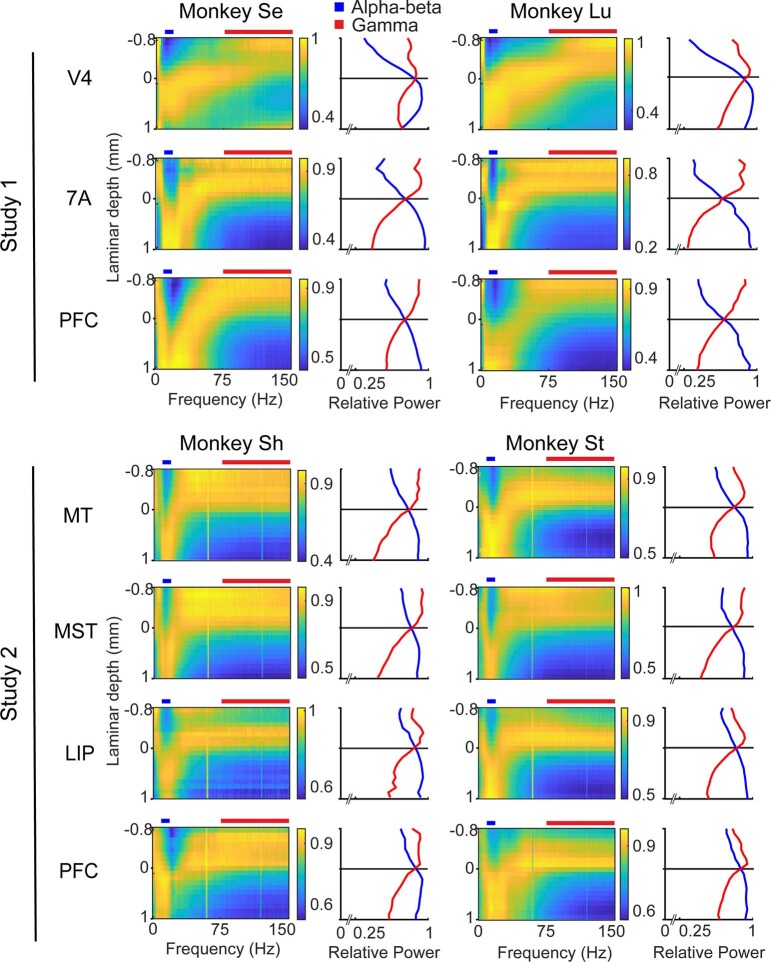
Spectrolaminar patterns as identified by FLIP. Across-probes average relative power maps (left) and average alpha-beta (blue) and gamma (red) relative power (right) for each area, monkey, and study. The selection of probes with identifiable spectrolaminar pattern, and the alignment of all probes by their cross-over channel, were performed by FLIP and were fully automated. The quality of the average spectrolaminar patterns is comparable or superior to that obtained with the manual identification method (Fig. 2).

We compared the performance of FLIP to our manual spectrolaminar pattern identification method. We found that a clear spectrolaminar pattern was identifiable in 64% of the probes using FLIP compared to the 61% identified with the manual method. We then compared the quality of the spectrolaminar patterns identified by FLIP and the manual method. We applied FLIP to our raw data and then obtained area-averaged relative power maps by repeating the population analyses used with the manual method in Fig. 2. Probes deemed identifiable by FLIP were included and aligned by the automatically identified crossover channel. The resulting area-averaged spectrolaminar patterns (Extended Data Fig. 5) were similar to those obtained with the manual method (Fig. 2). Our results indicate that, without user input, FLIP successfully identifies the spectrolaminar patterns obtained from laminar probe recordings, locates the three major physiological landmarks and uses them to provide the location of the recording channels with respect to cortical layers.

### Robustness of the spectrolaminar motif

**Fig. 6:**
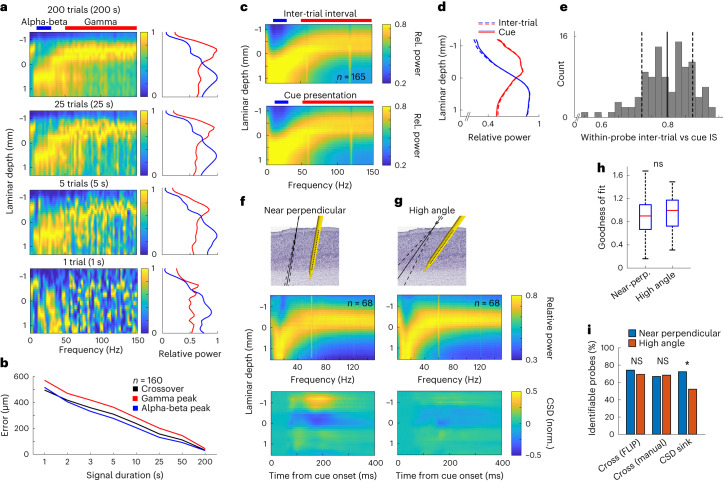
Robustness of the spectrolaminar pattern. **a**, Quality of spectrolaminar pattern as a function of signal duration. Relative power maps (left) and mean alpha-beta and gamma power bands (right; blue and red, respectively) for an example probe were obtained from varying durations: 200 s, 25 s, 5 s and 1 s. **b**, Error in localization of mean crossover, gamma peak and alpha-beta peak (determined by FLIP; Methods) estimated from signals of varying duration, with respect to the estimate from the entire recording session (>200 s). **c**, Mean relative power map across probes during inter-trial interval (top) and during cue presentation period (bottom). **d**, Mean relative power across probes in the alpha-beta (blue) and gamma (red) bands during inter-trial interval (dashed lines) and cue presentation (solid lines). **e**, Distribution of IS between inter-trial interval and cue presentation periods among individual probes. Mean ± s.d. is shown as vertical solid and dashed lines, respectively. **f**,**g**, Mean relative power maps (middle) and mean CSD maps (bottom) across probes with near-perpendicular insertion angles (**f**) and probes with high angles (**g**). Top panels illustrate mean probe insertion angle (solid line) ± s.d. (dashed lines). **h**, Mean *G* values for near-perpendicular (*n* = 68, minimum 0.16, first quartile 0.67, median 0.90, last quartile 1.09, maximum 1.68) and high-angle (*n* = 68, minimum 0.31, first quartile 0.72, median 1.00, last quartile 1.17, maximum 1.49) probe subpopulations (unpaired *t*-test, *P* = 0.56). **i**, Percentages are shown for near-perpendicular (blue) and high-angle (red) probes with identifiable relative power crossover (using automatic FLIP or manual methods) and identifiable CSD sink (manual). Proportions of identifiable probes were not significant for FLIP (two-sided chi-square test, *P* = 0.4152) or manual crossover detection (*P* = 0.8124), but CSD sink identification was significantly lower for high-angle probes (*P* = 0.001972). *, significant difference (chi-square test, *P* < 0.005); NS, not significant.

**Extended Data Fig. 6:**
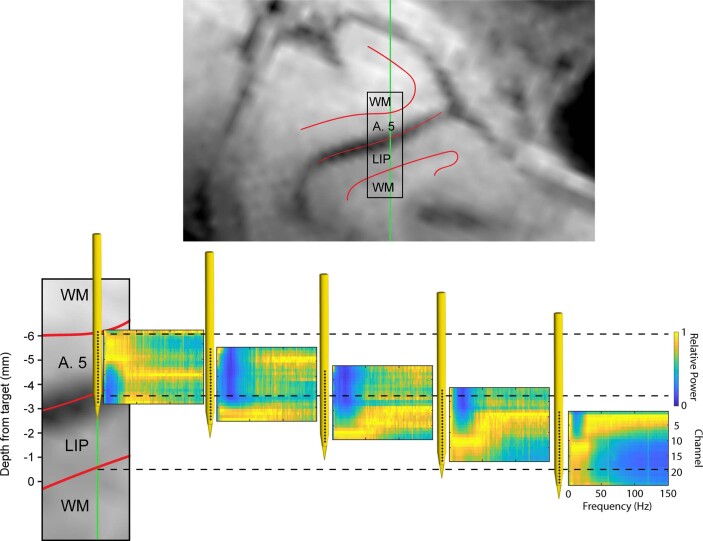
Transformation of relative power map during probe implantation in the cortex. Top panel: structural MRI section showing probe trajectory (green) across cortical layers in areas 5 and LIP. Bottom panel: Magnification of black rectangular region above, and corresponding relative power maps recorded at various probe depths. An inverted spectrolaminar pattern appears when the probe crosses the layers of Area 5 in a deep-to-superficial direction. Subsequently, an upright pattern appears gradually as the probe crosses the layers of LIP in a superficial-to-deep direction. The last map was acquired with the probe in the same position as the previous, but after waiting 1 hour; the cortex appears to relax after having dimpled during penetration, creating the illusion that the probe moved deeper. Examining the spectrolaminar patterns in close-to-real time during probe implantation provides the experimenter with a more precise method to track the probe position with respect to cortical sheets/layers than using previously-acquired MRI images for guidance. WM, white matter.

Next, we examined the robustness of the spectrolaminar motif in various recording and analysis conditions. First, in recordings made during probe insertion, we showed that the spectrolaminar motif was apparent as a probe entered, traversed and exited gray matter. The relative position and orientation of the pattern were indicative of the probe’s location relative to different cortical sheets (Extended Data Fig. 6). Second, we showed that the spectrolaminar motif was reliably identifiable within a few seconds of recording, as short as 5 s (Fig. 6a). On average, less than 25 s of data was required to generate a spectrolaminar pattern with less than 200 μm error in estimation of the crossover, gamma peak and beta peak locations. With 50 s of data, error in these metrics was approximately 100 μm (Fig. 6b).

Third, we showed that the spectrolaminar motif was observed both in the presence and in the absence of sensory stimulation—that is, during both the inter-trial interval and visual stimulation task periods (Fig. 6c,d). With respect to the visual stimulation period, the mean spectrolaminar pattern was highly similar in the inter-trial interval (IS = 0.99), in the fixation period (IS = 0.99) and in the delay period of the working memory task used in study 1 (IS = 0.9802) (all IS values were significantly higher than expected by chance; *P* < 0.001, permutation tests comparing IS in original versus channel-shuffled data, *n* = 1,000 shuffles). Therefore, the spectrolaminar motif is an omnipresent cortical state. The spectrolaminar patterns between the inter-trial interval and cue presentation were also similar at the single probe level (Fig. 6e; mean IS = 0.80, s.d. = 0.076).

**Extended Data Fig. 7:**
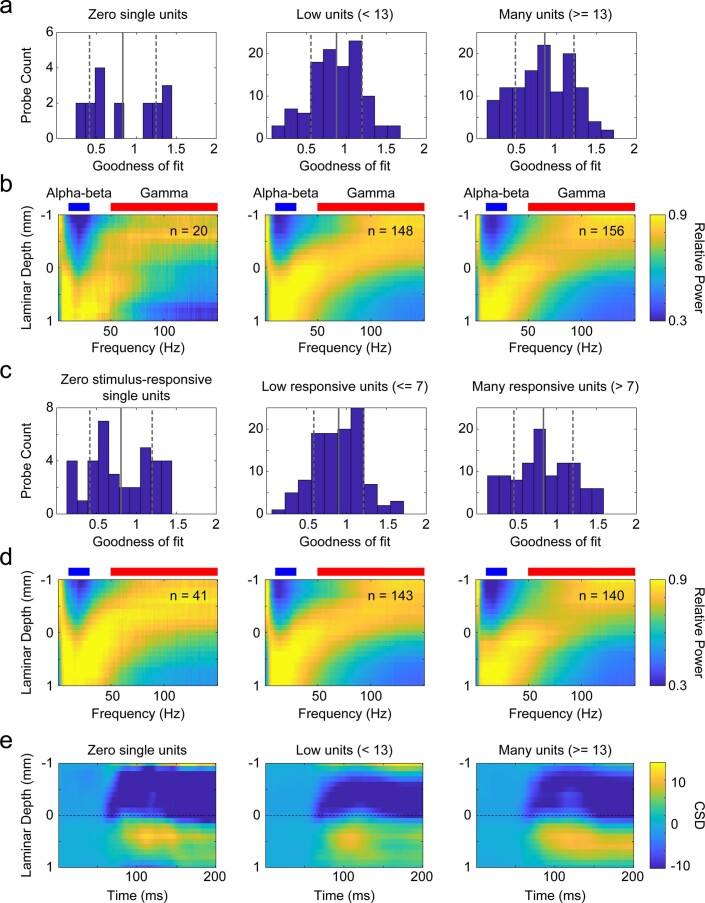
Relationship between spectrolaminar pattern/current source density sink and presence of single units in recording. We examined how robust the spectrolaminar pattern was to several recording quality metrics such as the number of single units detected and whether units on a probe had visual responses above baseline firing rate. We split our data from Study 1 into probes with zero units (n = 20), probes with low number of units (0 > number of units > 13, n = 148) and many units (>= 13 units, n = 156). These three subpopulations were examined for percentage identifiable crossover and goodness of fit value. Based on the FLIP algorithm, 17 (85%) of zero-unit probes had an identifiable crossover, 111 (75%) low unit probes had an identifiable crossover, and 120 (77%) of many unit probes had an identifiable crossover, and these differences in proportion were not significantly different (chi-square test, P > 0.05). (**a**) Goodness of Fit values for the population of probes recordings for which the number of isolated single units was zero (left), low (< 13, middle panel), and high (≥ 13 units, right panel). Absolute goodness of fit value distributions were not statistically different between subpopulations (zero unit probes 0.8302 mean +/ 0.4169 SD; low unit probes 0.8755 mean +/ 0.3201 SD; many unit probes 0.8517 mean +/ 0.3668; P(zero vs low) = 0.6014, CI = −0.2174 to 0.1268, t-stat = −0.5212, df = 126; P(zero vs many) = 0.8239 CI = −0.2128 to 0.1696, t-stat = −0.2229, df = 135; P(low vs many) = 0.6014 CI = −0.1133 to 0.0658, t-stat = −0.5231, df = 229; two-sample t-test). (**b**) Mean spectrolaminar maps for the different sub-populations of probes. The Image Similarity values were similar between subpopulations IS(zero vs low) = 0.5802; IS(zero vs many) = 0.5375; IS(low vs many) = 0.7603). We split our data from Study 1 into probe recordings where the number of stimulus-responsive units was zero (n = 41), low (0 < responsive units <= 7, n = 143), and high (> 7 responsive units, n = 140). Again, we found that the spectrolaminar pattern was present in about the same proportion across these groups. Based on the FLIP algorithm, 36 (88%) of zero responsive unit probes had an identifiable crossover, 109 (76%) low responsive unit probes had an identifiable crossover, and 103 (74%) of many responsive unit probes had an identifiable crossover (chi-square test, P > 0.05). *(***c***)* Goodness of Fit values for the population of probes where the number of stimulus-responsive units was zero (left panel), low (middle panel), and high (right). Absolute goodness of fit value distributions were not statistically different between subpopulations (P(zero vs low) = 0.1415; P(zero vs many) = 0.6969; P(low vs many) = 0.1598; unpaired t-tests). (**d**) Mean relative power maps were similar between subpopulations: IS(zero vs low) = 0.7411; IS(zero vs many) = 0.6112; IS(low vs many) = 0.8071. (**e**) Relationship between quality of CSD maps and single unit presence. Mean CSD maps across probe recordings where the number of single units was zero (left), low (middle), or high (right). The three probe groups had qualitatively similar patterns of sinks/sources.

Fourth, we showed that the spectrolaminar motif was present when the angle of the probe with respect to the cortical sheet was close to perpendicular (Fig. 6f) or more oblique (Fig. 6g). The spectrolaminar pattern was highly similar between low-angled and high-angled probes. This was quantified by a high IS value (0.92) and similar *G* values obtained by FLIP (Fig. 6h; unpaired *t*-test, *P* = 0.56). Low-angled and high-angled probes had a similar percentage of identifiable spectrolaminar patterns by FLIP (Fig. 6i; chi-square test, *P* = 0.42) and manual identification (Fig. 6i; chi-square test, *P* = 0.81). These findings suggest that the spectrolaminar motif is robust to various probe insertion angles. Fifth, we showed that there was no significant relationship between identification of the spectrolaminar motif as a function of the total number of single units isolated in the probe recording (Extended Data Fig. 7a,b; chi-square test, *P* = 0.60) or of the number of units with visual stimulus responses above baseline firing rate (Extended Data Fig. 7c,d; chi-square test, *P* = 0.17).

### Comparison of CSD to the spectrolaminar motif

It is well established that there is another pattern of neural activity known to map onto the cortical laminar architecture: CSD^20^. CSD shows the temporal dynamics of current sources and sinks after sensory input. In visual cortical areas, numerous studies have shown that, after the presentation of a visual stimulus, a current sink first occurs approximately in layer 4 and then travels toward more superficial and deeper layers^15,20,21,36–38^. Because this appears to be a common phenomenon across visual cortical areas, CSD has been used as the only established method to date to estimate the location of cortical layers in laminar electrophysiological recordings: the early CSD sink is used to estimate the location of layer 4. This raises at least two important questions. First, which of the two laminar patterns of activity—CSD or the spectrolaminar motif—is more ubiquitous across cortex? Second, which pattern maps more accurately onto the laminar anatomical cortical motif?

To address these questions, we obtained CSD as a function of time and channel depth for each probe in the datasets of study 1 and study 2 and estimated the channel at which the early sink occurred after the onset of visual stimulation. An identifiable early CSD sink (Methods) was present in 51% of the 810 probes recorded in study 1 and study 2. This was lower than the percentage of probes with an identifiable spectrolaminar motif (61% with manual identification, 64% with FLIP and 81% with vFLIP—see next subsection). This suggests that the spectrolaminar motif is more robustly present across the cortex than the CSD pattern.

**Fig. 7:**
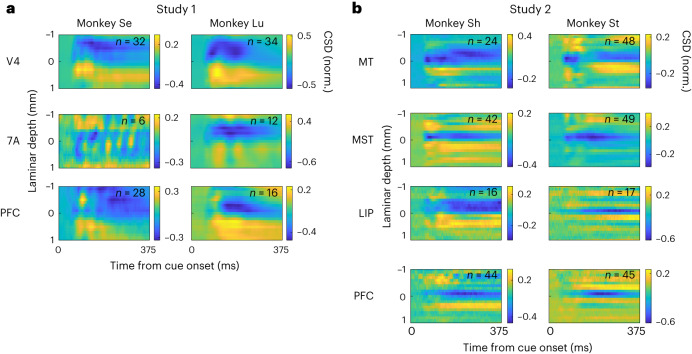
Comparison of CSD laminar patterns among cortical areas, monkeys and studies. **a**,**b**, Mean CSD maps across probes from each area and monkey in study 1 (**a**) and study 2 (**b**). Current sinks are negative (blue), and sources are positive (yellow). The CSD values of each probe were normalized by the peak negative value. Laminar position 0 (*y* axis) is the position of the first identifiable current sink.

**Extended Data Fig. 8:**
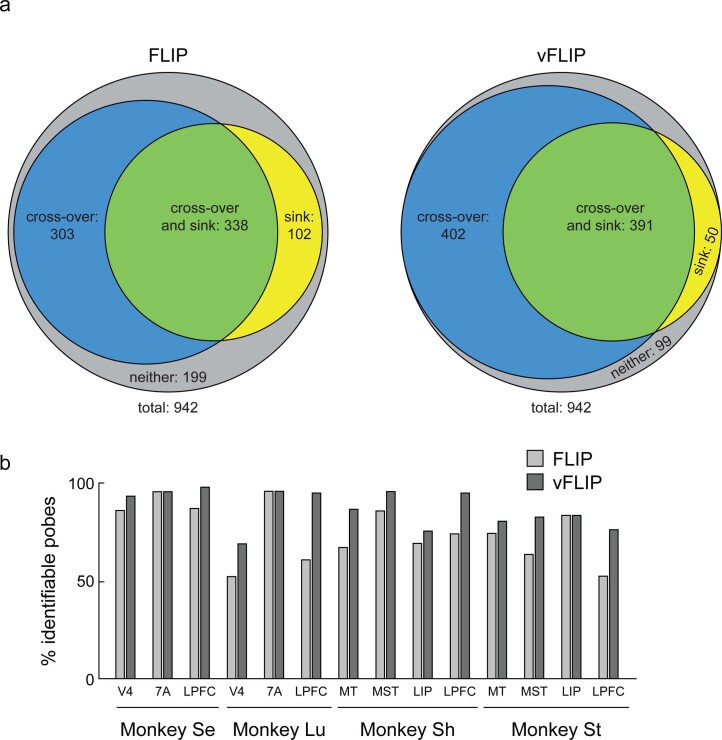
Comparison of spectrolaminar pattern identification by FLIP and vFLIP. (a) Venn diagram showing the percentage of probes with identifiable CSD early sink and/or identifiable spectrolaminar pattern by FLIP (left) or vFLIP (right). (b) Percentage of probes from each brain area with a spectrolaminar pattern identifiable by FLIP or vFLIP.

Among probes showing an identifiable early CSD sink, we next examined how preserved the CSD pattern was within and between cortical areas, monkeys and studies. For each probe recording showing a clearly identifiable CSD sink, we centered the CSD time-by-depth map at the early sink channel. We then averaged all probe CSD maps from each cortical area for each monkey in each study (Fig. 7a,b). Although the early CSD sink was largely visible in the mean CSD maps, the characteristics of the early sink, including duration, intensity and laminar thickness, appeared to vary markedly among areas, monkeys and studies (Fig. 7a,b). CSD sinks were more identifiable in visual areas V4, MT and MST compared to higher-order areas LIP and LPFC (Extended Data Table 1; chi-square test, *P* < 0.0001). This indicates that stimulus-induced CSD sinks are not a ubiquitous feature of laminar recordings, especially for higher-order cortex. This dissociation between visual regions and higher-order areas was not observed for identification of the spectrolaminar motif (Extended Data Fig. 8b).

**Extended Data Fig. 9:**
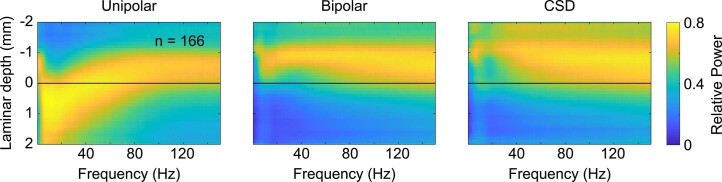
Across-probes mean unipolar (left), bipolar (middle), and CSD-derived (right) relative power maps. Left: Relative power maps were computed on a unipolar LFP referenced to an outer guide tube (as in Figs. 1–3). Middle: Data was first locally referenced to a bipolar montage by subtracting one electrode from its immediate neighbor along the probe. Subsequently, power was computed, and then the relative power across channels was calculated. Right: We first computed the CSD, then power, and then relative power across channels. The laminar depth of all maps is shown with respect to the alpha-beta/gamma cross-over channel from the unipolar data. We computed the spectrolaminar pattern based on the CSD (and, for completeness, the bipolar signal, see Methods) to determine whether in the spectral domain, CSD could provide better spatial estimates of the position of layer 4 than in the time domain. In contrast to the unipolar spectrolaminar maps, the bipolar and CSD spectrolaminar maps contained less features and peak relative power occurred in the superficial layers across frequencies. To determine whether the CSD/bipolar spectrolaminar maps contain more anatomical information compared to the unipolar spectrolaminar maps, we measured the distance between ‘power drop-off’ and layer 4. Power drop-off is the laminar depth at which CSD/bipolar relative power is equal to 0.6 (this often results in multiple intercepts, the value closest to layer 4 was used, see Methods). The mean distance from the CSD power drop-off to layer 4 across all areas was 96 μm (±126 μm SEM) and the distance from bipolar drop-off to layer 4 across all areas was 125 μm (±122 μm SEM). In contrast to the distance from the unipolar alpha-beta/gamma crossover to layer 4 across all areas (46 μm mean ±51 μm SEM). Both the CSD and bipolar drop-off to layer 4 distance metrics were more variable than the unipolar alpha-beta/gamma crossover (Ansari-Bradley test, P < 0.01 for unipolar vs. CSD, P < 0.01 for unipolar vs. bipolar). In general, visualization of the bipolar or CSD spectrolaminar pattern are complimentary to unipolar pattern, especially as they appear to demarcate the borders of superficial cortical layers. However, unipolar spectrolaminar pattern offers a more complex profile, including directionality. That is, if a probe is inserted into a deep region, far from the cortical surface, unipolar spectrolaminar power would provide more information, such as whether the gray matter is upright or inverted (L1-to-L6 or L6-to-L1; for example, area MST is inverted in our recordings, see Fig. 1b). Bipolar and CSD gamma drop-off were less informative since they do not provide directionality. Manual curation and comparison with unipolar crossover are required to determine the location of L4 in reference to bipolar or CSD relative power. Overall, bipolar and CSD spectrolaminar patterns are useful complements that could be plotted in addition to unipolar maps and are especially useful in identifying superficial layers. However, unipolar spectrolaminar patterns provide more information in identifying layer 4.

Thus far, electrophysiological studies in primates have used the CSD early sink as a standard electrophysiological marker to map layer 4. We, therefore, examined whether layer 4 was mapped more accurately by the CSD early sink or the alpha-beta/gamma crossover. We identified the channel location of the first identifiable CSD sink in the recordings from the electrolytic lesion sessions and determined its location with respect to the histologically defined location of layer 4 (Fig. 4c,d and Extended Data Fig. 3). We found that the median laminar position of the early CSD sink was 4.0, the same as that of the alpha-beta/gamma crossover (Fig. 4e). However, the 95% CI of the mean laminar position of the CSD early sink across probes (between 3.6 and 4.67, *n* = 14) was significantly larger than that of the crossover (between 3.82 and 4.45, *n* = 24), suggesting that the crossover more reliably mapped onto layer 4 than the CSD early sink (Fig. 4e; non-parametric Ansari–Bradley test for identical variance, *P* = 0.04). This difference in variability was also present when we randomly subsampled the number of probes to equate the number of probes providing alpha-beta/gamma crossover and CSD early sink (non-parametric randomization test for equal s.d. of the two distributions, *n* = 1,000 randomization, *P* < 0.05). The spectral power characteristics of the CSD signal fared no better than time domain CSD in providing anatomical accuracy of layer 4 (Extended Data Fig. 9).

Next, we showed that the presence of CSD sink patterns is highly sensitive to the probe insertion angle. The mean CSD pattern was much less visible for high-angled probes than for near-perpendicular probes (Fig. 6f,g). Comparing the mean patterns of near-perpendicular versus high-angled probes, IS between CSD sink patterns (IS = 0.24) was lower than that between spectrolaminar patterns (IS = 0.92) (Fig. 6f,g). Furthermore, the percentage of probes with identifiable CSD sinks was lower for high-angled probes than for near-perpendicular probes, and this difference was significant (Fig. 6i; chi-square test, *P* = 0.002). To determine whether CSD sink identifiability or crossover identifiability contribute to variability in probe angle, two-way ANOVA was performed. CSD sink identification (*P* = 0.0163), but not crossover identification (*P* = 0.1516), was a significant predictor of probe angle. High-angled probes were less likely to have an identifiable CSD sink. There was no significant interaction effect. Finally, to test whether the absence of CSD sink patterns could be related to data quality, we analyzed probes with a large number of isolated single units versus few. We found no relationship between the quality of CSD and the number of isolated single units (Extended Data Fig. 7e; chi-square test, *P* = 0.22).

### Comparison of the spectrolaminar motif across species

Although all mammalian species have a six-layer cortical motif, the laminar distribution of different cell types^39^ and their connectivity^40,41^ are different across species. These anatomical differences may imply cross-species differences in the laminar patterns of LFP power. The spectrolaminar motif observed in the macaque could be absent, present but qualitatively different or the same in other species. To examine this, we analyzed and compared laminar cortical recordings from macaque (942 probes), marmoset (54 probes), human (three probes) and mouse (291 probes) (Methods).

**Fig. 8:**
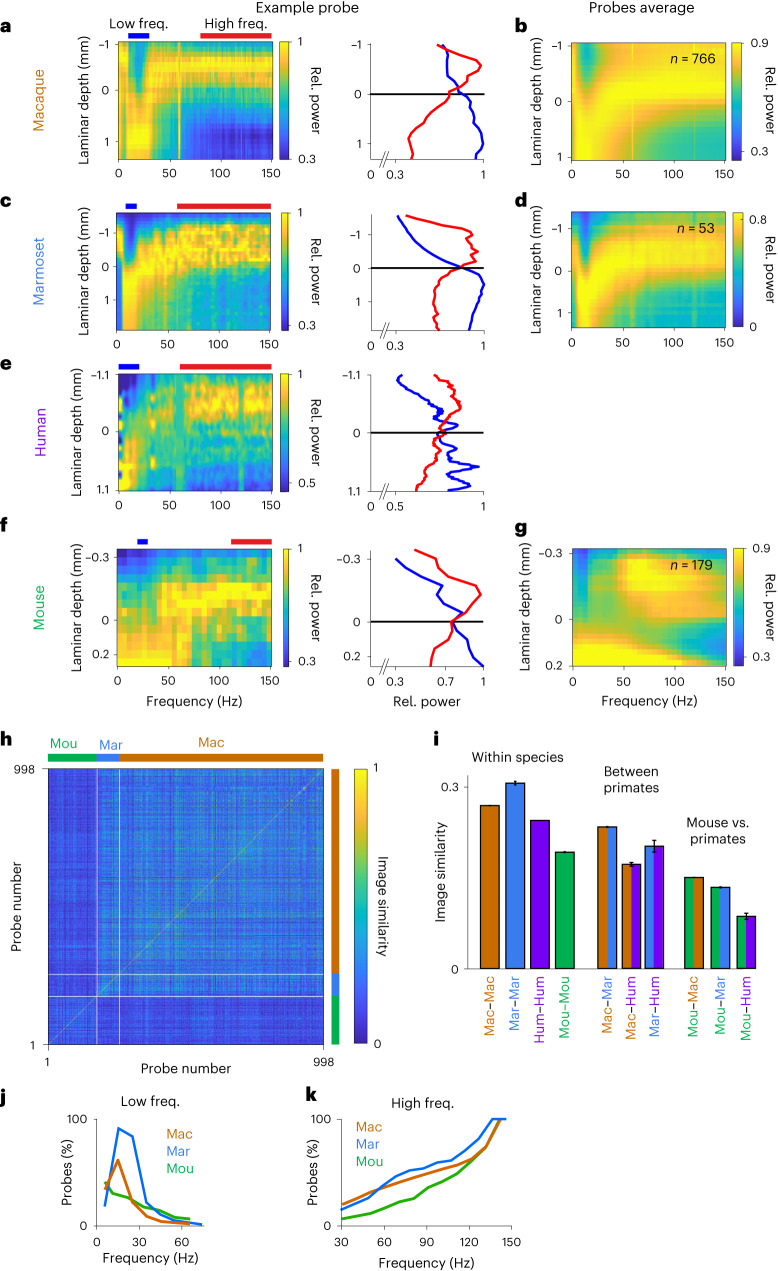
Comparison of the spectrolaminar pattern across species. **a**,**c**,**e**,**f**, For each species (macaque, marmoset, human and mouse), relative power map of an example probe (left) and corresponding mean relative power as a function of laminar depth in the low (blue) and high (red) frequency ranges of maximal laminar power gradients (right). **b**,**d**,**g**, For each species, mean relative power map across identifiable probes. **h**, Matrix of IS values (color scale) comparing the relative power maps between each pair of probes (color pixel) across macaque, marmoset and mouse datasets. Probes are sorted by species, separated by white lines. **i**, Mean IS (±s.e.) across all probe pairs within and between species. For each species comparison, IS values for all probe pairs are displayed in **h**, and the number of probe pairs is the product of the numbers of probes from both species (shown in **b**,**d**,**g**; for human, three probes). **j**,**k**, Percentage of probes from each species for which each frequency bin was included in the optimal low (**j**) or high (**k**) frequency ranges of vFLIP. For high-frequency ranges (**k**), the vFLIP algorithm did not explore frequencies below 30 Hz. (Note: for human, some population analyses could not be performed due to low sample size.)

In marmoset, as in macaque, relative power was organized by an increasing deep-to-superficial gradient of gamma power and an increasing superficial-to-deep alpha-beta power gradient (Fig. 8a,c). Of the three human probe recordings, two showed a clear spectrolaminar pattern similar to macaque and marmoset (Fig. 8e). In the mouse, relative power maps only partially resembled the spectrolaminar pattern seen in the three primate species above (Fig. 8f). Although most macaque/marmoset probes showed laminar power gradients that were most marked at gamma and at a relatively narrow frequency range around alpha-beta, the gradients in mouse probes were present across broader and more variable ranges of frequencies. To identify the spectrolaminar pattern in mouse recordings given this variability, we developed vFLIP, a frequency-variable version of FLIP that scans the entire frequency range up to 150 Hz for optimal lower-frequency versus higher-frequency bands that have opposing laminar power gradients (Methods).

vFLIP successfully identified the spectrolaminar pattern in 81% (766/942) of macaque probes, compared to 64% with FLIP and 61% with manual identification (Fig. 2b and Extended Data Fig. 8). vFLIP also identified the pattern in 53 of 54 marmoset probes. In the mouse, vFLIP identified the pattern in 62% (179/291) of the probes, significantly less than macaques and marmosets (chi-square tests, *P* < 0.001). We calculated the mean relative power map across all identifiable probes of each species aligned by their crossover channel. The mean map was very similar between the macaque and marmoset (Fig. 8b,d) but differed substantially in the mouse (Fig. 8g). Due to the frequency variability among mouse relative power maps, the spectrolaminar pattern appeared largely diffuse in the average map. These results suggest that the spectrolaminar motif is less preserved and more variable in the mouse compared to the other species.

We then performed IS analysis to compare the relative power maps of all probe pairs within and between species (Fig. 8h and Methods). Mean IS across probe pairs was higher for within-species than between-species pairs (Fig. 8i; unpaired *t*-test, *P* < 0.001). Mean IS for within-species probe pairs was significantly lower for mouse than for macaque and marmoset (Fig. 8i; unpaired *t*-tests, *P* < 0.001), indicating that there was more variability in the spectrolaminar pattern between probes in the mouse than in the non-human primates. Overall, IS values of probe pairs within non-human primates (macaques and marmosets) formed a square of higher values than those of mouse probe comparisons (Fig. 8h). Quantitatively confirming this, mean IS for between-species probe pairs was higher among all three primate species (macaque, marmoset and human) than between mouse and each of the primates (Fig. 8i; unpaired *t*-test, *P* < 0.001). These results suggest that the spectrolaminar pattern is more preserved between primates and more distinctive in the mouse.

We reasoned that the distinctiveness of the pattern in the mouse was partly due to differences in the frequencies at which the power gradients were present compared to the primate species. To quantify this, we examined the optimal low-frequency and high-frequency ranges of all probe recordings as identified by vFLIP (that is, those with maximal laminar power gradients). Across probes, the distribution of optimal low-frequency ranges showed a peak between 10 Hz and 30 Hz in the macaque and marmoset but was more evenly distributed and lacked this peak in the mouse (Fig. 8j). Compared to the macaque and marmoset, there was a lower fraction of mouse probes with an optimal high-frequency range that included the low gamma frequencies (40–80 Hz; Fig. 8k). Our results indicate that low-frequency and high-frequency laminar power gradients characteristic of the spectrolaminar motif are present in all species studied but that the frequency ranges at which these gradients occur are more similar among these primate species as compared to the mouse.

## Discussion

### A ubiquitous spectrolaminar motif across primate cortex

We report a spectrolaminar motif in the primate cortex consisting of frequency-specific LFP power gradients across cortical layers. Using electrolytic markers and histology, we determined that the peak of gamma power is located in superficial layers 2/3; the peak of alpha-beta power is in deep layers 5/6; and the crossover point between them is in layer 4.

We showed that this spectrolaminar motif is preserved in all cortical areas studied, suggesting that it is a ubiquitous property of the cortex. We showed that spectrolaminar patterns were more similar within each area than between areas. This supports the idea that each area is constructed as a specific variation around a canonical microcircuit and raises the question of how these variations contribute to the functional specialization of each area.

It is well established that there are patterns of systematic variation in laminar anatomy^8,9^. Sensory areas tend to have the most differentiated lamination, whereas motor areas have the least. What all cortical areas have in common is the presence of distinct superficial layer (layers 1–3) versus deep layer (layers 5/6) compartments. In this study, we showed that the spectrolaminar motif is present in areas that are highly laminated (for example, areas V1, V3 and V4) as well as areas that are much less laminated (for example, area 6/PMd). This suggests that the presence of the spectrolaminar pattern does not depend on the degree of lamination of each area, and that, instead, it is reflective of the superficial versus deep layer compartmentalization common across all areas. This hypothesis can be tested by comparing these cortical areas to other laminated structures, such as the hippocampus.

We also found that the spectrolaminar pattern was present in the marmoset, human and mouse. The deep-layer alpha-beta and superficial-layer gamma power profiles were more similar among the three primate species (macaque, marmoset and human) than between these primates and mouse. We speculate that this may reflect a divergence in the pattern of laminar oscillatory mechanisms between different mammalian orders (that is, primates versus rodents). In the mouse, laminar power gradients in deep layers were observed in broader and higher frequencies than in the primates studied. These differences may be explained by differences in the lamination patterns of inhibitory interneurons between mouse and macaque. In V1, the density of parvalbumin-positive and calbindin interneurons peaks in layers 2–4 in macaques but in layer 5 in mice^39^. Parvalbumin-positive interneurons have been implicated in the generation of gamma band activity^42^. Therefore, the distinct cellular composition across layers may explain the spectrolaminar motif and its variations across species.

### Comparison of the spectrolaminar motif to CSD

Previous work emphasized that the CSD pattern of sinks and sources reflects the activation of a canonical microcircuit^15,20–22^. Current sinks would first appear in layer 4 and then spread to superficial and deep layers. If this canonical model of activation were ubiquitous, a clear layer 4 CSD sink should be present across cortex. However, qualitatively, we did not detect current sinks beyond the input layer (layer 4) in several areas (Fig. 7). In addition, by three independent and quantitative measures, CSD was less ubiquitous and more variable than the spectrolaminar motif. First, the spectrolaminar motif was identifiable in a higher percentage of recordings (61% with manual identification, 64% with FLIP and 81% for vFLIP) than the CSD sink (51%). The CSD sink was more readily identifiable in visual cortical areas but not as reliable in higher-order areas (Extended Data Table 1). Second, we compared the variability in the mapping of CSD early sinks and the spectrolaminar alpha-beta/gamma crossover to the same anatomical reference point—the center of layer 4. CSD was more variable in its mapping of layer 4 compared to the spectrolaminar pattern (Fig. 4e and Extended Data Fig. 9). Third, the spectrolaminar motif was robust to a wider range of probe angles than CSD (Fig. 6f–i).

### Mechanisms and functions of the spectrolaminar motif

The precise neuronal mechanisms that generate the different oscillatory components of the spectrolaminar motif remain unknown. One possibility is that there is an epicenter for the generation of gamma rhythms (and perhaps also theta rhythms) in superficial layers. In addition, there may be a generative mechanism for beta rhythms in deep layers^25^ or in the interactions between deep and superficial layers^10,11,32,43^. Supporting this idea, previous studies in cortical slice preparations from rats showed evidence for the origins of gamma oscillations in layer 3 (ref. ^44^) and beta oscillations in layer 5 (refs. ^25,45^) or in the interactions between superficial and deep layers^25^. Whether these mechanisms are also present in vivo remains to be examined.

Superficial to deep layer volume conduction has been proposed to explain why alpha-beta power appears more powerful in superficial layers when calculated on bipolar or CSD signals^13,46^—the opposite pattern that we observed in the unipolar LFP referenced to the top of cortex. Recent biophysical modeling, however, suggests an alternative interpretation: an alpha-beta power peak in deep layers in the LFP (referenced to the top of cortex), together with a superficial CSD power peak (as observed by previous studies^13,46^ and in Extended Data Fig. 9), can both be modeled by considering the elongated cell bodies of deep-layer pyramidal neurons that receive synaptic inputs into apical dendrites in superficial layers as well as near the cell body in deep layers^47,48^. This modeling work suggests that the alpha-beta-generating circuitry includes both superficial and deep layers and is more spatially extended than the gamma-generating circuitry. To further understand the circuitry that generates alpha-beta and gamma rhythms, it will be necessary to perform more detailed studies that examine distinct types of inhibitory interneurons as well as where (on apical versus basal dendrites) they synapse onto pyramidal neurons.

CSD sinks and sources remained for decades as the only known activity pattern reflective of the canonical microcircuit, revealing a characteristic sequence of laminar current flows. The spectrolaminar pattern had been observed in some reports^5,10,12,15,18,24–28^, but its generality was questioned in others^11,28^. By performing extensive data collection from 14 macaque cortical areas combined within one analysis methodology, the present results establish the spectrolaminar motif as a ubiquitous cortical property. The spectrolaminar motif is, thus, the second known functional correlate of the laminar anatomical motif. Other electrophysiological features based on phasic modulation between fields and spikes^12,49^ and the power exponent of the LFP are also now beginning to emerge^50^. Importantly, this suggests that the canonical microcircuit also works via layer-specific neuronal oscillations and their power/phase characteristics and that such mechanisms play a fundamental role in cortical function^5^.

Anatomically, superficial layers provide the strongest feedforward cortico-cortical output, and deep layers provide the strongest feedback output^51^. Superficial versus deep layers form separate compartments^15^ that serve feedforward and feedback processing, respectively^5,52^. This superficial-layer feedforward and deep-layer feedback pattern is much more pronounced for long-range connections that span multiple hierarchical areas^53^. This implies that areas farther apart in the hierarchy are more functionally asymmetric compared to areas that are close together in the hierarchy. This has been tested with Granger causality applied to LFPs in macaque monkeys^18,31^, humans^33^ and mice^30^ and in simulations^32^. Our observations of a preserved spectrolaminar motif are consistent with these studies.

### Advantages and applications of FLIP and vFLIP

Historically, CSD was the only available method for laminar identification in electrophysiological recordings. In the present study, we developed an alternative method—a fully automated FLIP. FLIP offers many advantages over CSD. First, the functional landmarks in the spectrolaminar motif are identifiable in a higher percentage of probe recordings, particularly when the recording probe angle is not close to perpendicular to the cortex. Second, FLIP uses the spectrolaminar patterns, which are more generalizable and consistent across cortical areas, monkeys and studies than the CSD patterns. Third, layer 4 can be more accurately identified by the spectrolaminar pattern than by the CSD early sink. Fourth, the spectrolaminar pattern spans more layers and contains more physiological reference landmarks (gamma peak, alpha-beta peak and alpha-beta/gamma crossover) than CSD. Fifth, due to its low signal-to-noise ratio, CSD typically requires many trials of repeated stimulation. In contrast, the spectrolaminar motif can be identified in 5–25 s of data (Fig. 6a,b) collected without sensory stimulation or behavior (Fig. 6c–e). Fifth, in contrast to CSD, FLIP is fully automated and requires no user input beyond the laminar data (Fig. 5).

Besides FLIP, we also created a frequency-versatile version called vFLIP. vFLIP allowed successful identification of the spectrolaminar pattern in mouse and marmoset and in a larger fraction of macaque probes than FLIP. We hope that, in the future, as knowledge of the spectrolaminar pattern expands, frequency-based methods for layer identification will continue to improve. Therefore, the algorithms that we shared are meant to continue to evolve. Furthermore, it may be possible to improve the accuracy of layer localization methods by combining complementary information from spectrolaminar, CSD and neuronal spiking patterns.

The fact that FLIP is fully automated and fast opens the doors for the development of many applications. By continuously determining a probe’s location with respect to cortical layers in close-to-real time, FLIP may be used to guide probe placement during implantation (Extended Data Fig. 6). Moreover, probe placement could become fully automated and unsupervised by allowing the output of FLIP to perform closed-loop control of a computerized microdrive. In medicine, these methods may improve surgical implantation of probes in patients with epilepsy (for pre-surgical screening^54^), Parkinson’s disease (for deep brain stimulation^55^) and paralysis (for brain–machine interface systems^56^). Lastly, some neurological and psychiatric disorders are associated with abnormal beta oscillatory patterns (in Parkinson’s disease^57^) and abnormal gamma oscillatory activity (in schizophrenia^58^ and Alzheimer’s disease^59^). If these abnormalities result in atypical spectrolaminar patterns, measuring such patterns may help in understanding and eventually treating these disorders.

### A spectrolaminar framework for cortical electrophysiology

We think that a fundamental long-term goal in neuroscience should be to build a generalized cortical theory that explains how all cortical areas may accomplish a wide variety of functions via minor variations of the same theme—the canonical microcircuit. Given the laminar nature of this microcircuit, it will be crucial to establish laminar recordings as the common practice across electrophysiological studies of the cortex. One longstanding impediment to this goal in primate studies is that the cortex has been almost exclusively investigated either anatomically, through histological analyses in postmortem tissue but without access to neuronal activity, or electrophysiologically, by recording neuronal activity in behaving animals but without access to the anatomy. The ubiquity of the spectrolaminar motif and its relationship to the anatomical layers offers a unique opportunity to bridge the anatomical and electrophysiological approaches. By applying FLIP or vFLIP, electrophysiological signals can be mapped onto spectrolaminar space. The spectrolaminar approach will allow all electrophysiological studies of the cortex to use a common anatomical laminar reference as well as a common functional reference in the frequency domain. This standardized approach will lead to a better understanding of the specific roles of individual layers in cortical computations. The similarities (and differences) in these computations across areas will be key to unraveling the mechanistic principles of the canonical microcircuit.

## Methods

### Experimental model and subject details

Four adult rhesus macaques (*Macaca mulatta*) and one adult bonnet macaque (*Macaca radiata*) were used in this study. Five total macaque monkeys exceeds the field-specific standard of 2–3 animals per study (for example, refs. ^10–12,14,15^). Study 1 used two female rhesus macaques: monkey Se (6 years old and 5.0 kg) and monkey Lu (17 years old and 10.5 kg). In study 2, we used two male rhesus macaques: monkey Sh (9 years old and 13.7 kg) and monkey St (10 years old and 12.1 kg). Recordings in area V1 were performed in one additional male bonnet macaque (monkey Bo, 14 years old and 7.5 kg). The animals were housed on 12-h day/night cycles and maintained in a temperature-controlled environment (80 °F). All procedures were approved by the MIT/Vanderbilt Institutional Animal Care and Use Committee (IACUC) and followed the guidelines of the MIT/Vanderbilt IACUC and the US National Institutes of Health.

### Behavioral training and task

Monkeys sat in a primate chair inside a testing booth. Monkeys Se and Lu (study 1) were seated 50 cm away from a 24-inch LCD monitor with a 144-Hz refresh rate (ASUS). Monkeys Sh and St (study 2) were seated 57 cm away from a 27-inch LCD monitor with a 120-Hz refresh rate (Acer). Monkey Bo was seated 57 cm away from a 24-inch VIEWPixx /3D monitor with a 120-Hz refresh rate. Eye tracking was performed using an EyeLink 1000 system at a 1,000-Hz sampling rate in study 1, an EyeLink 2 system at a 500-Hz sampling rate in study 2 and an EyeLink 2 system at a 1,000-Hz sampling rate for the V1 study.

Using positive reinforcement, we trained monkeys to perform various tasks. For this study, we analyzed data only from the task periods before and during the initial stimulus presentation; other task details were irrelevant to this investigation. Monkeys in study 1 and study 2 were trained to fixate a point at the center of the screen (fixation window radius: 2–3 visual degrees for monkeys Se and Lu; 2.6 visual degrees for monkeys Sh and St) for a duration of 1 s and were then presented a cue stimulus. The cue stimulus was a naturalistic image in study 1 (chosen from three possible images) and a moving full-screen random dot surface in study 2 (5 dots/deg^2^; 0.15-deg dot diameter; 10.9-deg/s dot speed). We used repeated full-screen white flashes in the V1 study. For the main power and current source density analyses, we used the times in the task that were consistent across studies: we analyzed the period from 500 ms pre-cue to 500 ms post-cue.

### Electrophysiological recordings

All data were recorded through Blackrock headstages (CerePlex M), sampled at 30 kHz, band-passed between 0.3 Hz and 7.5 kHz (1st-order Butterworth high-pass filter and 3rd-order Butterworth low-pass filter) and digitized at 16 bit, 250 nV/bit. All LFPs were recorded with a low-pass 250-Hz Butterworth filter, sampled at 1 kHz and AC coupled.

In monkeys Se, Lu, St and Sh, we implanted a custom-machined PEEK or carbon PEEK chamber system with three recording wells. In monkeys Se and Lu, the recording chambers were placed over visual/temporal, parietal and lateral prefrontal cortex. In monkeys St and Sh, three recording chambers were placed over right parietal cortex and left and right lateral prefrontal cortex; in both prefrontal chambers, we additionally performed a durotomy and implanted a transparent silicon-based artificial dura. Monkey Bo was implanted with a 20-mm chamber over V1 and affixed with dental acrylic and ceramic screws. For monkeys Se, Lu, St and Sh, we obtained an anatomical MRI scan (0.5-mm^3^ voxel size) and/or computed tomography (CT) scan to extract the bone and co-register the skull model with the brain tissue. Chambers were placed to allow recording access to the primary areas of interest. Chambers for monkeys Se and Lu were additionally designed to have an optimal angle for perpendicular recordings relative to the cortical folding in areas V4, 7A and LPFC. For monkeys St and Sh, two chambers were designed to optimally cover LPFC (including posterior portions of areas 8Ad/v, 9/46d/v and 45 in right and left cortical hemispheres, one chamber per hemisphere) and one to access LIP, MT and MST at the most perpendicular angle possible (Fig. 1b). After the recording chambers were implanted, MRIs were taken with the recording grid in place and filled with water, which created a marker to co-register each possible recording grid probe trajectory with the animal’s anatomy and to confirm trajectories that were as close to perpendicular as possible.

We recorded a total of 213 sessions with laminar probes (monkey Se: 38; monkey Lu: 29; monkey Sh: 54; monkey St: 82; monkey Bo: 10). In each session, we inserted 1–6 laminar probes (‘U probes’ or ‘V probes’ from Plexon) into each recording chamber with 100-µm, 150-µm or 200-µm inter-site spacing and 16, 24 or 32 total contacts/channels per probe. This gave a total linear sampling of 3.0–3.1 mm on each probe. For all monkeys, the recording reference was the reinforcement tube, which made metallic contact with the entire length of the probe (total probe length from connector to tip ranged between 70 mm and 120 mm). When probes contained noisy channels (mean power greater than 2 s.d. above the mean of all channels, typically occurring in less than 5% of all channels), data for these channels were replaced with interpolations based on nearest neighbors before analysis.

For the original analyses included in Figs. 2, 5, 6 and 7 and Extended Data Figs. 1, 2, 5 and 8, we included *n* = 810 probe recordings from areas V4, 7A and LPFC in study 1 and areas MT, MST, LIP and LPFC in study 2. In subsequent analyses included in Fig. 8 and Extended Data Fig. 8, we increased the sample to *n* = 942 probe recordings by including additional recordings. In the analyses shown in Extended Data Fig. 1, we included probes from the areas with highest sample sizes: LPFC, V4, LIP, MST and MT (*n* = 838). The number of laminar probe recordings used in this study far exceeds the standard sample sizes used in most electrophysiological studies in non-human primates^10,11,13,15,18,21,24,27–29^. No statistical methods were used to predetermine sample sizes. To determine the mapping between CSD sinks and the spectrolaminar pattern, additional datasets were acquired, during which electrolytic lesions were performed (for more information, see the ‘Electrolytic lesion approach’ subsection). For Fig. 4c, *n* = 8 probes were used for LIP. For Fig. 4d, *n* = 10 probes were used for LPFC. For Fig. 4e, all available data from all probes/areas (including areas LIP, LPFC, MST, V1 and PMd) were included (total *n* = 24 probes). Areas with a smaller sample size are shown in Extended Data Fig. 4 (*n* = 3 probes in V1, *n* = 2 probes in MST and *n* = 1 probe in PMd).

For analyses to determine the robustness of the spectrolaminar pattern and CSD to the number of isolated units recorded in each probe (Extended Data Fig. 7), we used a total of 324 probes from study 1. For analyses of the spectrolaminar pattern of CSD and bipolar-referenced data (Extended Data Fig. 9), these 324 probes from study 1 were filtered to exclude probes without an identifiable spectrolaminar pattern and with low *G* value measured by FLIP (|*G|* value < 0.6), resulting in 166 probes.

To show that the spectrolaminar pattern was present in additional areas (Fig. 3), additional data were acquired that were not a part of the original study 1 or study 2 (*n* = 7 probes in V1, *n* = 13 probes in MIP, *n* = 12 probes in PMd, *n* = 7 probes in DP, *n* = 4 probes in somatosensory area 5, *n* = 3 probes in V3, *n* = 2 probes in Tpt and *n* = 1 probe in TPO).

#### Probe insertion and laminar placement

For monkeys Se and Lu, we first punctured the dura using a guide tube. Then, we lowered the laminar probes through the guide tube using custom-built drives that advanced with a turn screw system (as previously described in refs. ^10,62^). To place the channels of the laminar probe uniformly through the cortex, spanning from the surface through the gray matter to the white matter, we used a number of physiologic indicators to guide our probe placement, as previously described. First, the presence of a slow 1–2-Hz signal, a heartbeat artifact, was often found as we pierced the pia mater and just as we entered the gray matter. Second, as the first channels of the probe entered the gray matter, the magnitude of the LFP increased, and single-unit spiking activity and/or neural hash became apparent, both audibly and visually, with spikes appearing in the online spike threshold crossing. Once the tip of the probe transitioned into the gray matter, it was lowered slowly an additional approximately 2.5 mm. At this point, we retracted the probe by 200–400 µm and allowed the probe to settle for 1–2 h before recording. We left 1–3 channels out of gray matter in the overlying cerebrospinal fluid. We also used structural MRI guidance to inform approximate insertion depth and used the above criteria to finalize probe placement. We used the same general probe insertion procedure in monkey Bo, except that we used a custom-made drive from Narishige.

For monkeys St and Sh in study 2, we used a similar probe insertion procedure, with the following differences. Probe insertion was controlled with an electronic Microdrive (NAN Instruments). The probe location was estimated by the Microdrive penetration depth with reference to structural MRI maps, and precise placement across the cortical sheet in the target area was guided by the appearance of multi-unit and single-unit spiking activity across probe channels. We then waited for 30 min to 1 h before recording to allow probes to settle. Offline, probe trajectory angles were extracted from MRIs using OsiriX software. Zero degrees is considered perpendicular to gray matter (that is, in plane with cortical columns).

#### Electrophysiological recordings in marmosets

Fifty-four recordings were performed across three common marmosets (*Callithrix jacchus*) in cytoarchitectural area 8a (*n* = 28 probes) and lateral intraparietal area (*n* = 26 probes). This *n* of both number of animals and number of penetrations per area exceeds a previous report with laminar recordings in marmoset monkeys^29^. Recordings were performed using Neuropixels probes spanning approximately 3.8 mm (two columns of 192 channels, vertically spaced by 20 µm). For recordings, ground and external references were bridged and also connected to a copper isolation chamber. To improve signal quality, an internal reference located at the tip was used. However, this introduced artifactual noise outside of the brain. To correct this, electrodes were re-referenced to LFP contacts outside of cortex. Data were downsampled to 1 kHz, and channels were interpolated as described in the ‘Electrophysiological data analysis’ subsection to allow for comparison between species.

#### Electrophysiological recordings in mice

We used a publicly available electrophysiological dataset collected with Neuropixels probes in mouse (*Mus musculus*) visual cortex from the Allen Institute. Experimental details can be found in Siegle et al.^63^. The number of laminar probe recordings was 291 sampled across *n* = 13 female and *n* = 45 male mice. The relevant data collection and post-processing details are provided below.

All neural recordings were carried out with Neuropixels probes^64^. The experimental rig was designed for six Neuropixels probes to be inserted into the following visual cortical areas: primary visual cortex (V1), latero-medial (LM), anterol-ateral (AL), rostro-lateral (RL), postero-medial (PM) and antero-medial (AM). The penetrations were approximately perpendicular to the cortical surface, included all cortical layers and reached deeper into subcortical structures (hippocampus, thalamus and other nuclei). The recording sites of the probes are oriented in a checkerboard pattern (Neuropixels 1.0) on a 70-μm-wide × 10-mm-long shank, with a vertical separation of 20 µm between channels. The signals from each recording site were split in hardware into a spike band (30-kHz sampling rate, 500-Hz high-pass filter) and an LFP band (2.5-kHz sampling rate, 1,000-Hz low-pass filter). Gain settings of 500× and 250× were used for the spike band and LFP band, respectively. For data storage, the LFP data were further downsampled to 1,250 Hz. All data are openly accessible via DANDI (https://dandiarchive.org/). Electophysiological data were collected while mice were presented with full-field flash stimuli. Visual stimulation consisted of a series of dark or light full-field images with luminance at 100 cd/m^2^, lasting 250 ms each and separated by a 1.75-s inter-trial interval.

The cortical region was identified by determining the cortical surface and white matter of each insertion based on physiological features. The cortical surface was estimated by a sharp transition in low-frequency LFP power, and the white matter depth was estimated based on the gap in the unit density distribution along the probe. Based on the number of channels showing signals that fell within the cortical sheet, the estimated average cortical thickness was 0.64 mm. This value was lower than the mean mouse cortical thickness (∼0.8 mm (ref. ^65^)), likely due to the pressure on the cortex by the insertion window to maintain stability.

The dataset was available in the form of LFP power as a function of frequency (bins of 5 Hz) in each trial. Channels were interpolated as described in the ‘Electrophysiological data analysis’ subsection to allow for comparison between species.

#### Electrophysiological recordings in humans

We analyzed publicly available laminar electrophysiological data obtained from three human participants (patient (Pt) 01–03) in a previous study^66^. Participants were patients undergoing brain surgical treatments for epilepsy or movement disorders. Data were acquired during anesthesia (Pt 01 and Pt 03) or in awake resting state (Pt 02) from the dorsolateral prefrontal cortex (Pt 01 and Pt 02) and the lateral temporal lobe (Pt 03) using a thicker variant of the Neuropixels 1.0 probe with 960 recording sites spanning 10 mm along the shank and arranged in a checkerboard pattern of four columns with approximately 20-μm inter-site spacing. Each probe was inserted into a superficial cortical location and traversed the cortical sheet. Needle electrodes located in muscle tissue nearby (scalp) served as ground and recording reference. In each recording, signals were acquired from a selection of 382 channels. LFP signals were sampled at 2.5 Hz and band-pass filtered between 0.5 Hz and 500 Hz. Additional methodological details are available in the original article^66^.

All data analyses were as described for the macaque. Adjacent channels were averaged and interpolated to 100-µm spacing for comparison with the macaque data. In Fig. 8e, the relative power map of the example probe is shown with all recorded channels, but, to improve visualization of the spectrolaminar pattern, we applied smoothing across neighboring channels (five-channel moving window) and normalized the power of all channels by the mean power across channels within the vFLIP optimal frequency range.

### Electrophysiological data analysis

#### LFP power analysis

LFP power analysis was performed on 1-s time windows (500 ms pre-stimulus to 500 ms post-stimulus). The stimulus was either the cue stimulus onset (study 1 and study 2) or the flash stimulus (for V1 recordings). Power analyses used the FieldTrip toolbox for MATLAB^67^. We used the function ‘ft_freqanalysis’ with the method ‘mtmfft’. This implements a multi-taper spectral estimate^68^. We used 2-Hz smoothing in the spectral domain. Power was calculated on individual trials and then averaged across trials. We then obtained the relative power maps for each probe separately as follows:

$${{Relative}\,{Power}}_{(c,f)}=\frac{{{Power}}_{(c,f)}}{\max [{{Power}}_{(1:{nchan},f)}]}$$

where *c* is each channel on the probe, and *f* is each frequency from 0 Hz to 150 Hz. For each probe, this resulted in a two-dimensional matrix, with channels on the *y* axis and frequency on the *x* axis. Thus, at each frequency, every channel had an intensity between 0 and 1. Values of 1 indicate the channel that had the highest power at that frequency. For each frequency band (delta-theta: 1–6 Hz; alpha-beta: 10–30 Hz; gamma: 50–150 Hz), we then averaged, at each channel depth, the relative power values across all frequency bins within the band’s range, to obtain relative power as a function of channel depth (Figs. 1f,i and 5b). For probe recordings in areas where the cortical sheet was inverted due to its anatomical position within a sulcus (that is, entering from deep to superficial), the channel depths were inverted for all results.

Notably, the above helped confirm that the spectro-laminar pattern was not an artifact caused by proximity to the cortical surface. First, some of the areas were embedded deep within a sulcus (for example, MT and MST). Second, depending on the lip within a sulcus, some areas were approached from superficial to deep layers and others from deep to superficial layers (for example, MST). The orientation of the spectro-laminar pattern matched the laminar orientation of the area (for example, probes in MST showed inverted patterns).

To quantify the robustness of the spectrolaminar pattern across different signal durations, we examined three metrics: crossover, gamma peak and alpha-beta peak. Differences in these metrics between short signal durations and the actual crossover value (mean ± s.d. error micrometers, from entire session data) were calculated over 100 iterations of randomly selected groups of trials from a subpopulation of 160 randomly selected probes (Fig. 6b; for example, one random trial for 1-s signal duration, two random trials for 2-s signal duration, etc.). To compare population relative power maps during inter-trial interval (0.5 s before fixation onset until fixation onset) and during cue presentation period (first 500 ms of cue presentation), we analyzed a subsample of 165 probe recordings. For the analysis of probes by insertion angle, we grouped probes by the angle of insertion (relative to perpendicular axis) into near-perpendicular probes (lowest 25th percentile of all angles; angles < 14.0°; *n* = 68) and high-angle probes (highest 25th percentile of all angles; angles > 26.6°; *n* = 68).

#### FLIP

The automatic FLIP was designed as a fast, computer-based, fully automated method to determine the laminar locations of all probe channels in any laminar electrophysiological recording using the spectrolaminar pattern from LFP signals. We performed pilot tests of several types of algorithms to automatically detect the spectrolaminar pattern and found that an algorithm that detected opposite gradients of gamma and alpha-beta power across layers using linear fits was the most generalized algorithm among those requiring short runtimes. The input to FLIP is a power map—a two-dimensional matrix of LFP power values with frequency bins in the *x* axis and channels in the *y* axis. The channels dimension was resized with interpolation so that 24 interpolated channels span the estimated cortical thickness. This procedure standardized the channel spacing to the scale of cortical thickness regardless of the species and the recording probe’s inter-channel distances, thus allowing for comparisons across studies and species.

FLIP first obtains the mean laminar relative power as a function of depth for specific subranges of the alpha-beta (10–19 Hz) and gamma (75–150 Hz) bands. These subranges were chosen because, across most of our probe recordings, the laminar power gradients were steepest and most easily identifiable at these subranges. FLIP then iterates across all possible ranges of channel depths *D*_*i*_ to *D*_*f*_, where ∣*D*_*f*_ *−* *D*_*i*_ ∣ is at least 7 (corresponding to 700 μm in the macaque dataset); at each range *d*, it normalizes the power of all channels at each frequency bin dividing by the channel with the highest power within *d*. Then, it fits linear regressions through the alpha-beta and gamma power across depth (Fig. 5b) and computes the *G* value defined below:

$$G={{s}_{\alpha \beta }R}_{\alpha \beta }^{2}* -{{s}_{\gamma }}^{2}{R}_{\gamma }^{2}* f,$$

where

$$f=0.04* .\left({D}_{f}-{D}_{i}\right)+0.72,$$

and ${s}_{\alpha \beta }$ and ${s}_{\gamma }$ are the signs of the slopes of the linear regressions with coefficients ${R}_{\alpha \beta }^{2}$ and ${R}_{\gamma }^{2}$. FLIP then finds the range *d* that maximizes *G*. This maximum *G* serves as a measure of the quality of the spectrolaminar pattern (Fig. 5c,e). *r* is the most likely candidate channel range to align with the cortical sheet. The regularization term *f* ensures that the *G* value for smaller ranges is not higher simply due to a lower number of channel data points to fit.

If both regression coefficients are statistically significant, and if the absolute value of *G* exceeds the threshold *G*_*t*_ = 0.265, the probe is classified as having an identifiable alpha-beta/gamma crossover pattern, and the channels at which the alpha-beta peak, gamma peak and alpha-beta/gamma crossover occur are used to map the locations of layers 2/3, 5/6 and 4, respectively (Fig. 5b). The sign of *G* indicates the orientation of the probe insertion with respect to the cortical layers: a positive *G* indicates a superficial-to-deep insertion, leading to an upright spectrolaminar pattern; a negative *G* indicates a deep-to-superficial insertion, leading to an inverted spectrolaminar pattern (Fig. 5c,e).

Additional notes:

1. FLIP identifies and selects the gamma and alpha-beta peaks that are nearest the superficial (*D*_*i*_) and deep (*D*_*f*_) limits of the range *r*, respectively (either inside or outside the range).
2. To obtain the optimal threshold *G*_*t*_, we ran linear discriminant analysis to find the *G* value that best discriminated between probes that were determined to be identifiable versus un-identifiable based on detailed manual scrutiny. *G*_*t*_ was the value that minimized the sum of false-positive and false-negative cases in the discrimination.
3. In almost all probe recordings with a spectrolaminar pattern, the alpha-beta relative power (*P*_*αβ*_) and gamma relative power (*P*_*γ*_) crossed over at only one channel. For the few probes with more than one crossover channel, the algorithm selected the most likely crossover channel (*c*) by the following procedure. First, only crossover channels within the selected channel range *r* were considered. Second, a crossover between the alpha-beta relative power (*P*_*αβ*_) and gamma relative power (*P*_*γ*_) implies that *P*_*αβ*_ > *P*_*γ*_ at depths below *c* and *P*_*γ*_ > *P*_*αβ*_ at depths above *c*. We, therefore, selected the crossover channel that maximized the following power difference (Δ*P*):
  $$\Delta {\rm\it{P}}={\int }_{{Di}}^{c}\left({\rm\it{P}}{{\alpha }}{{\beta }}-{\rm\it{P}}{{\gamma }}\right)+{\int }_{c}^{{Df}}({\rm\it{P}}{{\gamma }}-{\rm\it{P}}{{\alpha }}{{\beta }})$$
4. To obtain the percentage of FLIP-identifiable probes after destroying laminar information (Fig. 2b), we randomized the laminar position of channels in each probe 100 times and ran FLIP each time. For the percentage of vFLIP-identifiable probes, the randomization procedure was repeated four times (due to longer runtime). The percentages reported are the mean across randomizations (Fig. 2b, light gray bars).

#### vFLIP

In addition to FLIP, we designed vFLIP, a frequency-variable version of FLIP. Although FLIP is limited to analyzing the laminar power gradients of the alpha-beta band (10–19 Hz) and gamma band (75–150 Hz) subranges, vFLIP repeats the same analysis as FLIP at multiple combinations of lower-frequency versus higher-frequency ranges and selects the combination of frequency ranges and channel depth ranges that yields the highest *G*. vFLIP tests all combinations of lower-frequency versus higher-frequency ranges that meet the following criteria: (1) the upper boundary of the lower frequency range must be ≤70 Hz and lower than the lower boundary of the higher-frequency range; (2) the lower boundary of the higher-frequency range must be above 30 Hz; (3) because the superficial gamma band does not show an upper frequency boundary in the spectrolaminar pattern, the upper boundary of the high-frequency range is fixed at 150 Hz—the highest frequency that we analyzed; and (4) to prevent a large number of range combinations, all combinations are constructed with boundaries by discrete steps of 10 Hz (for example, 10–20 versus 40–150, 10–30 versus 60–150, etc.). These criteria were chosen based on the occurrence of different combinations of frequency ranges across the datasets of all species used in this study. In addition to the outputs of FLIP, vFLIP also outputs the boundaries of the lower-frequency and higher-frequency ranges that yield the highest *G*. These represent the frequency ranges showing the highest laminar power gradients in opposite directions. Although vFLIP is slower to implement than FLIP, it is particularly useful for recordings where the optimal gradients do not fall within the alpha-beta and gamma bands. This was the case for a large fraction of recordings in the mouse and for a small fraction of recordings in the macaque.

#### CSD

CSD for each channel along the probe was based on previous work^20^ and defined as:

$${CSD}\left(c\right)=-\sigma \frac{{V}_{c-2}-2{V}_{c}+{V}_{c+2}}{(2{s)}^{2}}$$

where *V* is the voltage recorded at channel *c*; *s* is the inter-channel spacing; and *σ* is the tissue conductivity. CSD values were normalized by first subtracting the baseline (the mean CSD value of 200 ms to 0 ms pre-cue/pre-flash) and then dividing by the s.e.m. across trials. This converted the CSD into a unit-less measure of *z*-score units. This can be interpreted as the *z*-score units of change from baseline and allows the raw *z*-score value to express a degree of statistical confidence that a source or a sink was significantly different from baseline.

#### IS analysis

To quantify and compare the degree of similarity between the relative power maps of probe recordings within or between cortical areas, monkeys and studies, we employed IS analysis, a metric to quantitatively determine the pixel-by-pixel similarity between two images^34^. The images are composed of pixels arranged along a two-dimensional matrix, where each pixel is defined by a value along a single scale. The resulting IS value ranges between 0 (completely dissimilar) and 1 (identical). The analysis was performed using the MATLAB function SSIM. In our study, the pixels of the two-dimensional images corresponded to the frequency-by-channel matrices of normalized power values—that is, the relative power maps—with a size of 1-Hz frequency bins by number of channels (resized as described for FLIP).

We compared the relative power maps within each cortical area and between pairs of areas. This was done for all combinations of area pairs within and between monkeys and studies. We randomly subdivided the probes from each area into two equally sized subsets and then averaged the relative power maps of all probes in each of the two subset of each area. We computed IS comparing the mean relative power maps between the pair of subsets within each area and between all four combinations of between-area pairs of subsets, leading to four between-area IS estimates. We repeated this probe grouping procedure five times, thus obtaining a total of five within-area IS values and 20 between-area IS values. Averaging across these values yielded a grand within-area IS and a grand between-area IS (Extended Data Fig. 2).

To quantify the similarity between the relative power maps across species, we performed IS analysis on the maps of all pairs of probes within and across the macaque, marmoset, human and mouse datasets. All relative power maps were aligned by the crossover channel determined by vFLIP. Resizing the channels dimension (as described for FLIP/vFLIP) standardized the channel spacing across species to the scale of cortical thickness of each species. The analysis of IS between species used the relative power maps obtained by vFLIP. Because the mouse LFP power dataset was available in frequency bins of 5 Hz, the data from the other species (binned by 1 Hz) were re-binned to match the mouse bin size. This ensured that relative power map matrices had the same frequency dimension across species. The use of 5-Hz binning led to a lower range of IS values compared to those obtained for the macaque data binned at 1 Hz (Fig. 2c). Therefore, comparisons between IS values are only meaningful within each of these two analyses (Fig. 2c or Fig. 8h,i), not between them. For all statistical comparisons of IS (within macaque and between species), data distributions were assumed to be normal based on qualitative assessment, but normality was not formally tested.

Note that our study design did not require randomization of experimental conditions for data acquisition. Furthermore, because all electrophysiological data analyses were performed automatically in MATLAB, they were not subject to experimenter bias and did not require blinding. The only analysis that depended on user input was the manual assessment of the spectrolaminar pattern, and the results were similar to those obtained with a fully automated analysis (FLIP).

### Electrolytic lesion approach

To elicit CSD profiles, 70-ms full-screen white flashes were visually presented to the monkey once every 500 ms while tracking eye position. This was repeated between 400 and 2,000 times before selecting target channels for electrolytic lesions. Data were aligned to flash onset, and then CSD and relative power profiles were generated. Parameters for electrolytic lesions were similar to previous experiments: monopolar monophasic negative 10–20 μA for 20 s^37,69^. Current return was through the animal headpost, located at the posterior section of the skull. Current was verified on an oscilloscope measuring the voltage across a 10-kOhm resistor in series with the stimulation circuit. Care was taken to ensure that the stainless steel shafts of the stimulating Plexon V probes were not grounded. In monkey St, three lesions were performed (A-M Systems, Isolated Pulse Stimulator, model 2100) per probe recording. The first lesion was placed at the most superficial channel that was clearly in brain (sharp increase in spiking or gamma band activity). The second lesion was placed at the probe channel representing the crossover of alpha-beta and gamma bands. The third lesion was placed at the deepest probe channel. In monkeys Sh and Bo, only two lesions were made per penetration: one lesion at the crossover and one at the deepest channel. This decision was made because the most superficial lesions in monkey St were not visible upon histology—possibly because the current path avoided brain tissue and exited the brain along the surface cerebrospinal fluid to reach the headpost current return. In monkeys St and Sh, electrolytic lesions were performed in LPFC, LIP, MST and premotor area 6. In monkey Bo, lesions were performed in V1 while delivering 70-ms flashes every 250 ms.

### Histology

#### Perfusion surgery

Animals were anesthetized with ketamine (7.5 mg kg^−1^ intramuscular) and dexmedetomidine (0.015 mg kg^−1^ intramuscular). To provide anatomical landmarks for the electrolytic lesion locations and to later inform the correct slicing plane, angel hair pasta noodles (∼1-mm diameter, 13–24 mm into brain) were inserted at the same angle as probe penetrations. In LPFC chambers, noodles were placed at medial and posterior coordinates of the chamber. In the parietal chamber, noodles were placed at medial and anterior chamber coordinates. Lethal sodium pentobarbital solution (40 mg kg^−1^ (intravenous) or greater) was started immediately after noodle placement. Perfusion surgery details were previously described^70^. In brief, the animal was perfused transcardially with 30% PBS, followed by 4% paraformaldehyde and, finally, 4% paraformaldehyde with 10% sucrose. Whole brain was stored in sucrose PBS until ready for sectioning and slicing. Brain was sectioned along the hemispheric midline and then cut into separate blocks for each chamber. These MRI-guided cuts were estimated to be in plane with noodle and probe penetration angles. Blocks were sliced at 40 μm on a freezing microtome, and every other section was stained for Nissl substance.

#### Imaging and block reconstruction

We imaged each Nissl section using a Zeiss Axio Imager.M2 microscope at an imaging magnification factor of ×1.25. This corresponded to a pixel-to-space conversion factor of 8 μm per pixel.

After imaging, we aligned the images to reconstruct the full tissue block in three dimensions. We loaded the anatomical images into a 3D slicer (https://www.slicer.org/ (ref. ^71^)) using the ImageStacks plugin^72^. Within this three-dimensional tissue block, we identified the noodle locations as well as the electrolytic locations that were pre-defined in a coordinate system aligned to the noodles.

#### Lesion identification and probe reconstruction

After the creation of the lesions and collection of associated electrophysical data, an individual coordinate system was established in each animal by the placement of two markers (angel hair pasta noodles). Two frames of reference were primarily used to locate lesion locations, the established coordinate system and a set slide order. Other anatomical features, such as the positioning of sulci and gyri, were used to determine positions of each electrolytic lesion in a tissue block within the 3D slicer. Once located, cortical lesions appeared as roughly circular marks of approximately 100–300-μm diameter stained a darker purple color compared to the surrounding tissue. In many cases, both or all three lesion marks per probe were identifiable, but, in other cases, one or no lesion marks were present. In cases where either no electrolytic lesions were identified or only one, we were not able to locate the laminar location of the recording probe. After identification of each cortical lesion location, we began virtual probe reconstruction—that is, determining probe channel locations with respect to specific cortical layers.

We reconstructed the probe locations by combining the known number of channels, lesion sites and inter-channel spacing. A virtual probe model was created in accordance with this information, and it was then scaled down using a global factor accounting for tissue shrinkage that occurred during the staining process. The distance between the coordinate markers was measured in the sections and compared to the known distance between them before staining. This resulted in a scaling factor of 0.87, meaning that the final imaged tissue was 87% the size of the real tissue, similar to shrinkage in previous reports^73,74^.

A higher number of aligned lesion locations per probe along with punctate and round lesion marks increased confidence of a successful alignment. Using the virtual probe location, electrophysiological data were assigned to each cortical layer. Peaks in the gamma and alpha-beta relative power bands (alpha-beta, 10–30 Hz; gamma, 50–150 Hz) as well as the crossover of alpha-beta/gamma relative power were identified, and their physical locations were analyzed relative to the cortical layers present. Measurements of the distance from the peaks and crossovers in power were taken in micrometer units from the center of cortical layer 4.

### Reporting summary

Further information on research design is available in the Nature Portfolio Reporting Summary linked to this article.

## Online content

Any methods, additional references, Nature Portfolio reporting summaries, source data, extended data, supplementary information, acknowledgements, peer review information; details of author contributions and competing interests; and statements of data and code availability are available at 10.1038/s41593-023-01554-7.

## Supplementary information

Reporting Summary

## Figures and Tables

**Extended Data Table 1:**
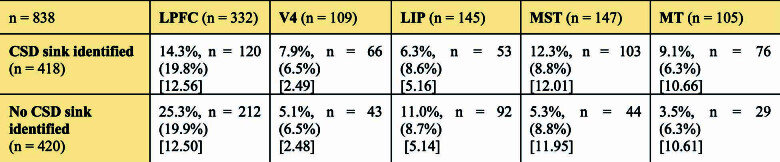
Chi-square test for relationship between CSD early sink identification and brain region

After calculation, values were converted to percentages. Actual values and percentages for each cell are shown. Expected percentages are in parentheses. Chi-square statistic for each cell is shown in square parentheses. Generally, more probes than expected had identifiable CSD sinks in visual areas V4, MST and MT, and fewer probes than expected had identifiable CSD in higher-order areas PFC and LIP.

## Data Availability

Normalized relative power and current source density for all probes used in study 1 and study 2 have been archived at the Dryad server: 10.5061/dryad.9w0vt4bnp. This includes raw LFP signals from two example laminar probes.

## Code availability

We created user-friendly MATLAB commands to implement FLIP and vFLIP. These have also been posted on the aforementioned Dryad server: 10.5061/dryad.9w0vt4bnp. Example MATLAB functions, scripts and instructions are provided therein to assist implementation of FLIP and vFLIP to the example datasets and oneʼs own laminar electrophysiological data.
