# Supplementary material for: A ubiquitous spectrolaminar motif of local field potential power across the primate cortex
Source: Nat Neurosci. 2024 Jan 18;27(3):547–60. doi: 10.1038/s41593-023-01554-7 (PMC10917659; doi:10.1038/s41593-023-01554-7)
Supplement: Supplementary file 1 — Reporting Summary [file 41593_2023_1554_MOESM1_ESM.pdf]

nature portfolio

Corresponding author(s): Diego Mendoza-Halliday, Andre M. Bastos

Last updated by author(s): Aug 14, 2023

Reporting Summary

Nature Portfolio wishes to improve the reproducibility of the work that we publish. This form provides structure for consistency and transparency in reporting. For further information on Nature Portfolio policies, see our Editorial Policies and the Editorial Policy Checklist.

Statistics

For all statistical analyses, confirm that the following items are present in the figure legend, table legend, main text, or Methods section.

n/a	Confirmed	
☐	☒	The exact sample size (*n*) for each experimental group/condition, given as a discrete number and unit of measurement
☐	☒	A statement on whether measurements were taken from distinct samples or whether the same sample was measured repeatedly
☐	☒	The statistical test(s) used AND whether they are one- or two-sided<br>*Only common tests should be described solely by name; describe more complex techniques in the Methods section.*
☐	☒	A description of all covariates tested
☐	☒	A description of any assumptions or corrections, such as tests of normality and adjustment for multiple comparisons
☐	☒	A full description of the statistical parameters including central tendency (e.g. means) or other basic estimates (e.g. regression coefficient) AND variation (e.g. standard deviation) or associated estimates of uncertainty (e.g. confidence intervals)
☐	☒	For null hypothesis testing, the test statistic (e.g. *F*, *t*, *r*) with confidence intervals, effect sizes, degrees of freedom and *P* value noted<br>*Give P values as exact values whenever suitable.*
☒	☐	For Bayesian analysis, information on the choice of priors and Markov chain Monte Carlo settings
☒	☐	For hierarchical and complex designs, identification of the appropriate level for tests and full reporting of outcomes
☒	☐	Estimates of effect sizes (e.g. Cohen's *d*, Pearson's *r*), indicating how they were calculated

Our web collection on statistics for biologists contains articles on many of the points above.

Software and code

Policy information about availability of computer code

Data collection	Eyelink 1000 and Eyelink 2 software (eye tracking); Blackrock Microsystems (electrophysiological data acquisition); Osirix software (MRI-guided probe trajectories).
Data analysis	Matlab 2017 and later versions / FieldTrip tooldbox (electrophysiological data analyses); 3D slicer/ ImageStacks (histological image reconstruction). Example code and data to implement VFLIP can be found at: https://doi.org/10.5061/dryad.9w0vt4bnp.

For manuscripts utilizing custom algorithms or software that are central to the research but not yet described in published literature, software must be made available to editors and reviewers. We strongly encourage code deposition in a community repository (e.g. GitHub). See the Nature Portfolio guidelines for submitting code & software for further information.

Data

Policy information about availability of data

All manuscripts must include a data availability statement. This statement should provide the following information, where applicable:

- Accession codes, unique identifiers, or web links for publicly available datasets
- A description of any restrictions on data availability
- For clinical datasets or third party data, please ensure that the statement adheres to our policy

An example dataset can be found on Dryad server: https://doi.org/10.5061/dryad.9w0vt4bnp. This includes normalized power and current source density information for hundreds of probes throughout macaque cortex and raw LFP signals from two example laminar probes. In addition, we have created user-friendly MATLAB commands to implement FLIP and VFLIP. These have also been posted on the aforementioned Dryad server. Example MATLAB functions, scripts, and instructions are provided therein to assist implementation of FLIP and VFLIP to the example datasets and your own laminar electrophysiological data.

Research involving human participants, their data, or biological material

Policy information about studies with human participants or human data. See also policy information about sex, gender (identity/presentation), and sexual orientation and race, ethnicity and racism.

Reporting on sex and gender	We analyzed publicly available laminar electrophysiological data obtained from three human neurological patients (Pt 01–03) in a previous study (Paulk et al., 2022). Complete information of the patients can be found in the original study. The complete dataset of the original study included seven female and two male patients. Neither sex nor gender were part of the study design.
Reporting on race, ethnicity, or other socially relevant groupings	Race, ethnicity and other social groupings were not part of the study design.
Population characteristics	Participants' mean age was 59 years old (range 34–75).
Recruitment	As described by the original study: Participants were neurological patients already scheduled for a craniotomy for concurrent clinical intraoperative neurophysiological monitoring or testing. Patients were contacted via phone and recruited for the study on a voluntary basis.
Ethics oversight	As described by the original study: All patients voluntarily participated after informed consent according to guidelines as monitored by the Massachusetts General Brigham (previously Partners) institutional review board at Massachusetts General Hospital (MGH).

Note that full information on the approval of the study protocol must also be provided in the manuscript.

Field-specific reporting

Please select the one below that is the best fit for your research. If you are not sure, read the appropriate sections before making your selection.

☒ Life sciences ☐ Behavioural & social sciences ☐ Ecological, evolutionary & environmental sciences

For a reference copy of the document with all sections, see nature.com/documents/nr-reporting-summary-flat.pdf

Life sciences study design

All studies must disclose on these points even when the disclosure is negative.

Sample size	Due to pooling of data from two independent studies, the sample size used for all main analyses (number of macaque probe recordings = 810) was larger than is common in non-human primate electrophysiological studies. (refs 10,11,13,16,21,24,25,28,29,30)
Data exclusions	For all major data analyses, no probe recording data was excluded. For analyses of the spectrolaminar pattern of CSD and bipolar-referenced data, we excluded probes without an identifiable spectrolaminar pattern and with low Goodness of fit measured by FLIP (|G| < 0.6).
Replication	All of the main findings in the study were replicated in datasets from two independent studies.
Randomization	We randomized the order of channels along the laminar dimension of the probe to test the randomization distribution of the FLIP algorithm.
Blinding	Using FLIP and vFLIP, spectrolaminar analyses were fully-automated and did not require user input. Therefore, no blinding was necessary.

Reporting for specific materials, systems and methods

We require information from authors about some types of materials, experimental systems and methods used in many studies. Here, indicate whether each material, system or method listed is relevant to your study. If you are not sure if a list item applies to your research, read the appropriate section before selecting a response.

Materials & experimental systems

n/a	Involved in the study	
☒	☐	Antibodies
☒	☐	Eukaryotic cell lines
☒	☐	Palaeontology and archaeology
☐	☒	Animals and other organisms
☒	☐	Clinical data
☒	☐	Dual use research of concern
☒	☐	Plants

Methods

n/a	Involved in the study	
☒	☐	ChIP-seq
☒	☐	Flow cytometry
☒	☐	MRI-based neuroimaging

Animals and other research organisms

Policy information about studies involving animals; ARRIVE guidelines recommended for reporting animal research, and Sex and Gender in Research

Laboratory animals	Rhesus macaque (Macaca mulatta), age range 6-17 years old.<br>Common marmoset (Callithrix jacchus), age range 22-38 months<br>Mouse (Mus musculus), age range P25-50.
Wild animals	No wild animal were used.
Reporting on sex	Macaques: 3 female, 1 male.<br>Marmosets:2 female, 1 male<br>Mice: 13 female, 45 male.
Field-collected samples	No field-collected samples were used.
Ethics oversight	Macaques: All procedures were approved by the MIT/Vanderbilt IACUC and followed the guidelines of the MIT/Vanderbilt Animal Care and Use Committee and the US National Institutes of Health.<br>Marmosets: All experimental methods described were performed in accordance with the guidelines of the Canadian Council on Animal Care policy on the care and use of experimental animals and an ethics protocol approved by the Animal Care Committee of the University of Western Ontario<br>Mice (as described in the original study): Mice were maintained in the Allen Institute for Brain Science animal facility and used in accordance with protocols approved by the Allen Institute's Institutional Animal Care and Use Committee.

Note that full information on the approval of the study protocol must also be provided in the manuscript.
